# Responses of mediterranean freshwater invertebrates to the fungicide difenoconazole across different macrophyte dominance conditions: A mesocosm study

**DOI:** 10.1007/s10646-026-03042-7

**Published:** 2026-02-18

**Authors:** Daniel Grillo-Avila, María Antón-Pardo, Javier Armengol, Eric Puche, Jesús Moratalla-López, José Francisco Palacios-Abella, Isabel López-Heras, Carlos Rochera, Antonio Picazo, Antonio Camacho, Andreu Rico

**Affiliations:** 1 Institut Cavanilles de Biodiversitat i Biologia Evolutiva, Universitat de València, C/ Catedràtic José Beltrán Martinez 2, Paterna (València), 46980 Spain; 2 IMDEA Water Institute, Av/ Punto Com, 2, Parque Científico Tecnológico de la Universidad de Alcalá 28805, Alcalá de Henares, Spain

**Keywords:** Pesticides, Freshwater communities, Mediterranean wetlands, Mesocosms, Macrophytes

## Abstract

Mediterranean wetlands are exposed to multiple environmental stressors, including chemical pollution from agricultural practices. Difenoconazole, an azole fungicide used in rice fields to control rice blast, may reach concentrations in Mediterranean wetlands that exceed toxicity thresholds for aquatic organisms. However, its long-term effects on freshwater communities are still poorly understood. This study evaluated the impact of difenoconazole on zooplankton and macroinvertebrate communities using outdoor freshwater mesocosms that simulated Mediterranean wetland conditions over 90 days. Two ecological conditions were tested: vegetated mesocosms containing *Myriophyllum spicatum* and non-vegetated mesocosms. Four exposure concentrations were applied twice, with a 14-day interval: control (0 µg/L), low (2 µg/L), medium (20 µg/L), and high (200 µg/L). Difenoconazole caused significant effects on zooplankton and macroinvertebrate communities at concentrations above 20 µg/L. Cladocerans and copepods were particularly sensitive, with some population reductions observed even at 2 µg/L. Among macroinvertebrates, the snail family Planorbidae showed declines at concentrations above 2 µg/L, while the freshwater shrimp *Dugastella valentina* was affected at concentrations exceeding 20 µg/L. The presence of macrophytes slightly mitigated pesticide effects by enhancing their dissipation from the water column. Indirect effects were also detected, including increased chlorophyll-a concentrations at the highest concentration, suggesting reduced zooplankton grazing pressure. Overall, the results indicate that the chronic threshold value for difenoconazole derived from species sensitivity distributions obtained from the literature is generally protective under semi-field conditions. However, peak exposures in rice fields and drainage ditches may significantly reduce the abundance of some invertebrate taxa under Mediterranean conditions.

**Supplementary Information:**

The online version contains supplementary material available at 10.1007/s10646-026-03042-7.

## Introduction

Chemical pollution is recognized as one of the primary drivers of biodiversity loss in Mediterranean coastal wetlands (Martínez-Megías and Rico 2022). These ecosystems are contaminated by a diverse range of chemical compounds, including pharmaceutical residues and agricultural pesticides (Amador et al. 2025; Barbieri et al. 2021; Martínez-Megías et al. 2024). Rice cultivation, a traditional agricultural practice in Mediterranean coastal wetlands, often relies on the use of fungicides due to the warm and humid conditions associated with this crop (Rodrigo et al. 2022; Zheng et al. 2013). Fungicides from the strobilurin and azole groups are the most employed in rice crops within the Mediterranean region (Amador et al. 2025). Among azole fungicides, the use of difenoconazole has significantly increased, replacing other fungicides in the same group, such as tebuconazole (Martínez-Megías et al. 2023). Monitoring studies conducted in the Albufera Natural Park (Valencia, Spain) have reported difenoconazole concentrations in surface waters ranging from 0.11 to 0.25 µg/L (Soriano et al. 2024a, b), while, in Malaysia, maximum concentrations of up to 170 µg/L have been found in water bodies near rice fields (Latiff et al. 2010). Furthermore, a modelling study has identified difenoconazole as one of the most hazardous substances for aquatic organisms in Mediterranean wetlands, with concentrations in rice paddies potentially exceeding 10 µg/L after application, and maximum exposure concentrations in drainage ditches and downstream water bodies ranging between 1 and 5 µg/L (Amador et al. 2025).

Azole fungicides, such as difenoconazole, inhibit the biosynthesis of ergosterol by affecting cytochrome P450 monooxygenases (CYPs), one of the most important enzyme classes present in all realms (Snyder 2000). Due to its mode of action, it may have toxic effects in a wide range of organisms, presenting biocidal effects (Zubrod et al. 2019). Sun et al. (2024) demonstrated that difenoconazole exerts chronic effects on growth and reproduction of freshwater invertebrates at concentrations that are two orders of magnitude lower that those exerting acute mortality. In the same study, these authors identified two crustaceans, the mysid shrimp *Americamysis bahia* and the cladoceran *Daphnia magna* as highly sensitive taxa, with long-term NOECs (No Observed Effect Concentrations) affecting survival and reproduction at 2.3 µg/L and 5.3 µg/L, respectively. As for vertebrates, difenoconazole has been demonstrated to exert effects on oxidative stress and organ damage, affecting reproduction (Nataraj et al. 2023; Wu et al. 2023). For example, Chen et al. (2023) found a NOEC of 1 µg/L for fertility in *Danio rerio* adults exposed to difenoconazole for 127 days.

Recently, Sun et al. (2024) proposed acute and chronic environmental threshold concentrations for difenoconazole based on the Hazardous Concentration for 5% of species (HC5) derived from Species Sensitivity Distributions (SSDs) constructed with laboratory toxicity data for primary producers, invertebrates and fish. They found that the HC5 for aquatic organisms due to acute exposure was 100 µg/L, while the HC5 due to chronic exposure was 0.96 µg/L. The derived chronic threshold falls within the range of chronic exposure concentrations (21-d time-weighted average concentrations: 0.5–1.5 µg/L) modelled by Amador et al. (2025) for drainage ditches in Mediterranean wetlands, which underscores the need for further research on the long-term effects of this compound on aquatic populations and communities.

Micro- and mesocosm studies provide valuable tools for evaluating the effects of contaminants on populations and communities, as well as their interactions with biotic and abiotic parameters within aquatic ecosystems (Macaulay et al. 2025). To date, the effects of difenoconazole on micro- or mesocosm systems have not been investigated. However, studies involving fungicides from the azole group suggest that aquatic communities and their trophic interactions can be significantly disrupted by exposure to these pesticides. For instance, Dimitrov et al. (2014) demonstrated that the azole fungicide tebuconazole alters the feeding behavior of the amphipod *Gammarus pulex*, a key detritivore. This effect was mediated through fungicide-induced shifts in the fungal community, which reduced the palatability of the food source and ultimately led to decreased consumption rates.

Numerous studies have highlighted the critical role of wetland conservation status in shaping the impact of additional stressors on aquatic biodiversity, thereby enhancing ecosystem resilience (Bereswill et al. 2014; Gregoire et al. 2009; Rodrigo et al. 2022; Turner et al. 2000). For instance, macrophytes serve as a natural defence mechanism against the harmful effects of pesticides by acting as physical barriers that reduce chemical exposure (Bouldin et al. 2005; Moore et al. 2009). Additionally, macrophytes provide a substrate for periphyton and offer food resources and shelter that are essential for a wide range of aquatic organisms (Carabal et al. 2024), creating habitats and refuges for species that are particularly vulnerable to chemical pollution (Brogan and Relyea 2015). For example, Grillo-Avila et al. (2024) demonstrated that freshwater mesocosms dominated by the submerged macrophyte *Myriophyllum spicatum* L. facilitated the establishment of benthic diatom populations. These mesocosms also supported larger macrocrustacean populations, such as *Dugastella valentina*, which were found to be more sensitive to the herbicide bentazone compared to the insect taxa that tend to dominate systems devoid of macrophytes. Recovery times for aquatic populations following pesticide exposure also vary by ecosystem type. In experiments with lambda-cyhalothrin, Roessink et al. (2005) observed faster recovery in ecosystems without submerged macrophytes, which were dominated by the dipteran larva *Chaoborus obscuripes*. Conversely, recovery was slower in macrophyte-rich ecosystems dominated by benthic macrocrustaceans like *Gammarus pulex*. The authors attribute this disparity to the shorter generation times and superior resistance and dispersal traits of *C. obscuripes*. Despite these insights, the influence of macrophyte dominance and overall trophic status on pesticide impacts in wetlands requires further investigations.

The objective of this study was to evaluate the long-term impact of difenoconazole on aquatic communities typical of Mediterranean wetlands under two contrasting ecological conditions: one characterized by the presence of submerged macrophytes dominated by *M. spicatum* L., and the other lacking such vegetation. The research was conducted experimentally using outdoor mesocosms with species assemblages typical of Mediterranean wetlands, exposed to different concentrations of difenoconazole. Furthermore, the experiment evaluated the relevance of proposed threshold concentrations of difenoconazole for safeguarding invertebrate populations and communities representative of the Mediterranean region. It was hypothesised that chronic exposure to difenoconazole may result in adverse effects on the structure and functioning of aquatic communities, thereby generating differential ecotoxicological responses depending on the presence of submerged macrophytes. The findings elucidate the distinct pathways through which difenoconazole impacts aquatic ecosystems and provide critical threshold concentrations to guide the management and conservation of Mediterranean coastal wetlands.

## Materials and methods

### Experimental design

The experiment was conducted in 24 mesocosms located at the Albufera Biological Station, which is located in El Palmar (Valencia, Spain) (Lat.: 39.315572; Long.: -0.319642). The PVC mesocosms utilised in the present study were the same as those employed by Grillo-Avila et al. (2024). Each mesocosm contained 10 cm of silty-clay soil from a riparian area (organic matter content 3–4%) and was filled with 1154 L of dechlorinated tap water. A preliminary phase prior to the experiment was conducted to inoculate the mesocosms with zooplankton and macroinvertebrate samples collected from nearby ponds and limnocrene springs (see Grillo-Avila et al. 2024 for further details). The inoculum samples were subjected to homogenisation in 30 L of dechlorinated water, after which they were added to each mesocosm (1 L per mesocosm). This procedure was carried out twice in September 2023. During both, the pre-experimental and experimental phases, water losses were compensated with the addition of dechlorinated water previously filtered through a 20 μm net.

In each mesocosm, baskets containing pebbles and traps filled with *Populus sp.* leaves and pebbles were incorporated, serving as substrates for macroinvertebrate sampling. Additionally, two mesh bags (mesh size 500 μm) with *Populus* sp. leaves were used to evaluate the effects of the fungicide on the decomposition rate of organic matter performed by microorganisms and small detritivores (< 500 μm).

In half of the mesocosms (*N* = 12), 66 shoots (22 bundles of 3 shoots, each 40 cm long) of the aquatic plant *Myriophyllum spicatum* were introduced (macrophyte treatment: M), while the remaining mesocosms (*N* = 12) were left without macrophytes (no macrophyte treatment: NM). The macrophytes were collected from springs with low contamination levels, washed with tap water, kept in a climate-controlled chamber for seven days, and rinsed again before being planted in the mesocosms. In the acclimation period, mesocosms were left to develop planktonic and macroinvertebrate populations. To minimize differences between replicates, water was occasionally redistributed among units of the same treatment (M or NM) with the aid of a water pump or buckets.

Four concentrations of the fungicide difenoconazole were tested within each of the two ecological conditions (M and NM): control (0 µg/L), low (2 µg/L), medium (20 µg/L), and high (200 µg/L), in triplicate. Difenoconazole was administered in two applications, with a 14-day interval between each application, considering a similar time interval between applications as used in rice fields. The first application was done on October 30, 2023 (Day 0), and the second on November 13, 2023 (Day 14), coinciding with the period when the rice fields are drained, after which there are some pesticide peaks in downstream water bodies due to the movement of water and the transport of suspended particles. The experiment had a duration of three months from the initial pesticide application. Sampling of zooplankton and macroinvertebrate communities was conducted on three occasions: Day − 7 (D-7, seven days before the initial application of difenoconazole), Day 30 (D30, one month after the first application), and Day 90 (D90, three months from the first application), similar to the experiment conducted by Grillo-Avila et al. (2024).

### Chemical application, sampling, and analytical verification

To obtain the stock solution, 9.2 g of MAVITA^®^ (containing 25% difenoconazole) were dissolved in 1 L of distilled water and maintained under stirring at 50 °C for 30 min to allow a complete dissolution, producing a concentration of 2.3 g a.i./L. Aliquots of 1, 10, and 100 mL of this stock were further diluted to 1 L, and the resulting solutions were added to the experimental tanks (1154 L) to establish the target levels of 2, 20, and 200 µg/L. After addition, each mesocosm was gently mixed with a wooden stick (different for each concentration to avoid cross-contamination) to ensure an even distribution of the compound in the water column. Difenoconazole concentrations were monitored on D0 (2 h post-application), D7, D14, D21, D30, and D45. Controls were sampled on D0 and D30 to confirm absence of (cross-)contamination. For each measurement, 5 mL subsamples were taken from a 5 L depth-integrated water sample, transferred to amber glass vials, and frozen at − 20 °C prior to analysis. Difenoconazole analyses were conducted following the method described in the Supporting Information file (Text S1 and Table **S1**, Supplementary Material). The Limit of Quantification (LOQ) of the method was 0.5 µg/L, and the Limit of Detection (LOD) was 0.05 µg/L. Finally, measured concentrations were expressed as percentages of the intended doses. Dissipation constants (k) were determined from the decline in exposure levels after the second application, applying a first-order kinetic model, and the half-life of difenoconazole (DT50) was computed as ln (2)/k.

### Physical and chemical parameters

Water quality parameters, namely temperature (°C), electrical conductivity (µS/cm), and dissolved oxygen (mg/L), were recorded on site with a WTW Multi 3410 multiparameter logger. pH was recorded in situ with a Crison Basic-20 pH-meter. Laboratory analyses of 5 L samples per mesocosm included soluble reactive phosphorus (SRP), total phosphorus (TP), nitrate (NO₃⁻), ammonia (NH₄⁺), alkalinity, and total suspended solids (TSS), applying APHA (2005) methods. Sampling took place on days 7, 30, and 90, relative to the first difenoconazole application.

### Phytoplankton assessment

Phytoplankton biomass was assessed using the chlorophyll-a concentration (Chl-a, µg/L) as a proxy, on D-7, D30 and D90 relative to the first fungicide application following Picazo et al. (2013).

### Submerged macrophytes

Submerged macrophyte cover was visually assessed on days 30 and 90 in each mesocosm. At the end of the experiment (D90), three biomass samples (area = 0.031 m² per mesocosm) were obtained with a 20-cm corer. Samples were stored in plastic bags, drained of excess water, and subsequently air-dried in the laboratory for 8–10 days. Dry weight (DW) of macrophytes per mesocosm was measured with a scale (Mettler PJ3000, with a resolution of ± 0.1 g). The relationship between vegetation DW and the percentage of vegetation cover was established by linear regression for the two different ecological conditions separately. The regression equation calculated was then utilised to estimate the biomass of the aquatic plants corresponding to D30 and D90 in the various mesocosms.

### Organic matter decomposition

The decomposition of organic matter by microbial and microinvertebrate activity was assessed by the introduction of two litter bags (mesh size 500 μm) introduced into each mesocosm and containing 10 g of *Populus* sp. leaves each. Prior to the introduction, the leaves were subjected to a drying process (40 °C for 48 h) and weighted. The bags were suspended from a wooden rod at a depth of 30 cm on D0, and one was collected on D30 and the other one, on D90. After collection, the bag content was dried (60 °C for 48 h) and weighted again to quantify the total decomposition percentage.

### Zooplankton

Zooplankton samples were collected on D-7, D30, D60, and D90. The additional sampling day (D60) was incorporated into the experimental design only for this taxonomic group due to its temporal variability and to better observe possible indirect effects. Six water samples from each of the mesocosm were collected using a PVC pipe (9 cm diameter, 95 cm length), and subsequently pooled into a bucket. A 5 L portion of this sample was filtered using a zooplankton net with 55 μm mesh size. Collected zooplankton individuals were fixed in Lugol’s iodine. Taxonomic determination of rotifers, cladocerans, and copepods was carried out with an inverted microscope (Leica DM IL LED) at 40–100x, and all specimens were counted. Copepod records were further subdivided into adult, copepodite, and nauplius stages. Results are presented as number of organisms per litre (ind/L).

### Macroinvertebrates

Macroinvertebrate samples were taken on D-7, D30 and D90 relative to the first fungicide application. Pelagic and benthic individuals were sampled collecting the pebble traps and leaf baskets and capturing individuals from the water column using a 500-µm net. The sample obtained from the net, basket and traps mixture was filtered using three sieves with mesh sizes of 1 cm, 1 mm and 250 μm and all the contents were placed on a tray. Each taxon was identified and counted by eye, and then the contents of the tray were returned to the corresponding tank. Sampling was carried out in ascending order of concentration to avoid cross-contamination. Abundances were expressed as individuals per sample.

### Data analyses

For macrophyte variables (cover, biomass, and relative growth) in the vegetation treatment, differences among difenoconazole concentrations were tested with one-way ANOVA.

For zooplankton and macroinvertebrates, species richness (S), total abundance (N), and Shannon diversity index (H′) were calculated for each sampling date. These indices, together with the abundance of the major invertebrates groups, were analysed using a two-way ANOVA, with fungicide concentration and macrophyte condition (M vs. NM) as factors. The same approach was applied to evaluate fungicide and vegetation effects on physicochemical parameters and organic matter decomposition. Normality and homoscedasticity were tested in all analyses, and in the cases when they were not met (abundance of zooplankton and macroinvertebrates), data were log(x + 1)-transformed.

For individual taxa and water quality parameters, no observed effect concentrations (NOECs) were estimated with the Williams test (Williams 1972) using the Community Analysis software v4.3 (Hommen et al., 1994). Effects were considered consistent and treatment-related only when the following criteria were met (Amador et al. 2024): (i) significance at *p* < 0.05 in the Williams test; (ii) sufficient organism density in the control to allow detection (≥ 3 individuals/sample for each macroinvertebrate taxa, ≥ 3 individuals/L for each cladoceran and copepod taxa, ≥ 10 individuals/L for each rotifer taxa); (iii) a monotonic dose–response pattern within the test range; and (iv) absence of the same kind of effects in the pre-treatment period. This multi-criteria approach was implemented to minimize false-positive evaluations for low-abundance species and to ensure the statistical robustness of the results.

Community-level responses of zooplankton and macroinvertebrates were further assessed with permutational multivariate analysis of variance (PERMANOVA; Anderson 2001), using Bray–Curtis similarity matrices built from log(x + 1)-transformed abundances. Analyses were run separately for each sampling date (999 permutations). When significant treatment effects were detected, pairwise comparisons were performed to establish community-level no observed effect concentrations (NOECs), defined as the highest concentration that did not differ significantly from the controls. Ordination by principal coordinates analysis (PCoA) was used to visualise treatment-related changes in community structure. Multivariate statistics were conducted with PRIMER v7 (PRIMER-E Ltd., Plymouth, UK).

## Results

### Exposure concentrations of Difenoconazole

The results of the difenoconazole analyses after the first application showed that the measured concentrations were, on average, 100% of the intended concentration in the treatment without macrophytes, and 95% in the treatment with macrophytes, indicating that there were a proper stock solution preparation and dosing of difenoconazole in the test mesocosms. After the second application, these values were 179% and 225%, respectively, indicating that there was concentration build-up (**Table ****S2**). No residues of the test compound were found in the controls.

**Fig. 1:**
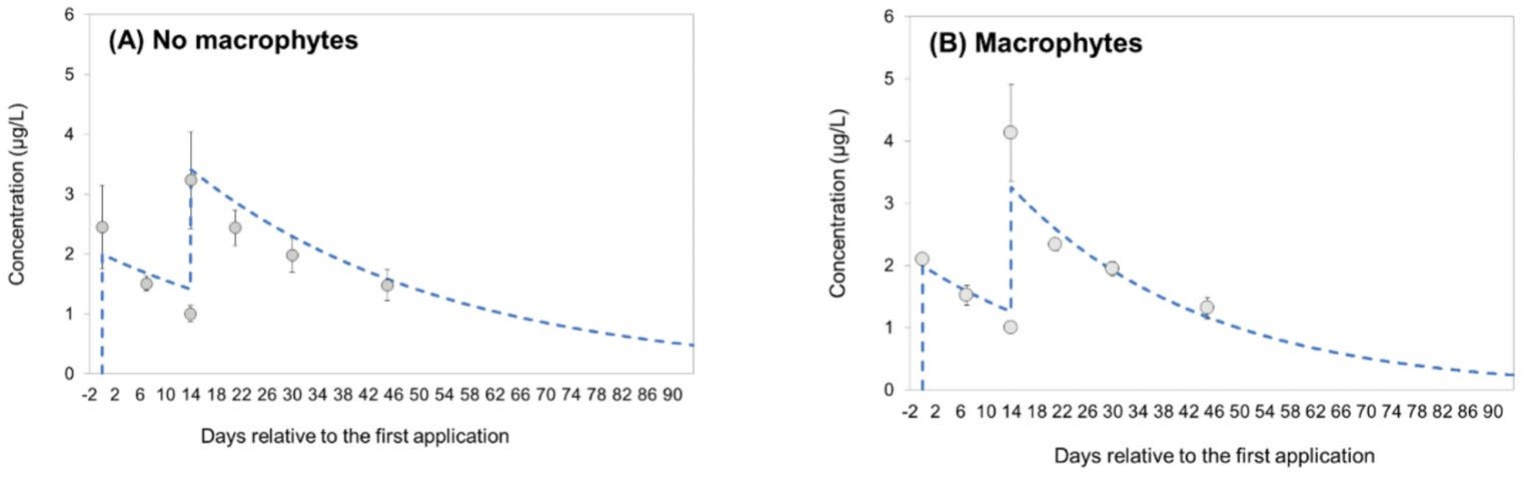
Measured concentrations of difenoconazole in the lowest treatment (2 µg/L) without (**A**) and with macrophytes (**B**). The circles show the measured concentrations of difenoconazole in the mesocosms (mean ± SD), while the dashed line indicates the modelled concentration based on first-order kinetics and the calculated dissipation coefficients (Table S3)

The DT50 of difenoconazole ranged between 28 and 68 days in the treatment without macrophytes (mean DT50: 43 days), and between 21 and 43 days in the treatment with macrophytes (mean DT50: 34 days; Table S3), showing that the dissipation of the test compound from the water column was faster in the treatment with macrophytes. The measured concentrations of difenoconazole in the lowest concentration treatment mesocosms (2 µg/L) are shown in Fig. 1, while a complete overview of the exposure concentrations is provided in **Table ****S2****.**

### Physical and chemical parameters

The mean water temperature was similar in both ecological conditions (14.10 ± 2.66 °C), with minimum values recorded on D30 (12.30 ± 0.36 °C) and maximum values recorded on D-7 (17.84 ± 0.32 °C; Table S4). The mesocosms with macrophytes exhibited a significantly lower conductivity (1209 ± 34 µS/cm) and alkalinity (1.13 ± 0.20 meq/L) on D30 and D90 in comparison to the mesocosms without macrophytes (1257 ± 35 µS/cm and 1.74 ± 0.44 meq/L, *p* < 0.05). In addition, the mesocosms containing macrophytes had significantly higher pH (9.08 ± 0.33; *p* < 0.05) and DO concentration (12.37 ± 0.24 mg/L; *p* < 0.01) on D30 and D90 than the values observed in the mesocosms without macrophytes (8.75 ± 0.35 and 11.45 ± 0.34 mg/L, respectively). A significant increase in nitrate concentration was observed at the highest difenoconazole concentration (200 µg/L) compared with the controls in both ecological conditions, with values of 27.23 ± 2.91 µM and 30.29 ± 1.79 µM on D30, and 28.28 ± 2.79 µM and 31.68 µM on D90, respectively. No significant effects related to the fungicide treatment were observed for the rest of the physico-chemical parameters measured in the experiment (Table S5** and **Table S6).

### Phytoplankton assessment

A slight, but statistically significant increase in chlorophyll-a concentration was found in the highest fungicide concentration (2.61 µg/L) compared to the controls (1.07 µg/L) in the mesocosm with macrophytes (**Table S4-S6**).

### Organic matter decomposition

The mean organic matter decomposition rate (**Figure ****S1**) was significantly higher on D30 in mesocosms with macrophytes compared to those without macrophytes (*p* < 0.01). On D90, however, no significant differences were found (*p* > 0.05). No significant effects on organic matter decomposition rates related to the fungicide concentrations were found (Table S5 and Table S6).

### Submerged macrophytes

No significant effects of the fungicide were observed on the *M. spicatum* cover and biomass on D30 and D90. Furthermore, no significant differences were observed in the relative growth rate of the macrophytes from D30 to D90 for the different concentrations (**Table S7 and Table S8**).

### Zooplankton community

Overall, the zooplankton community was composed of 38 taxa: 27 Rotifera, 9 Cladocera and 2 Copepoda. The most abundant organisms were juvenile copepod stages (nauplii and copepodites) of two species (*Acanthocyclops americanus* and *Copidodiaptomus numidicus*) and rotifers (*Keratella cochlearis*, *K. tropica*, *Polyarthra dolichoptera* and *Lecane closterocerca*). Significant differences in the abundance of zooplankton organisms were observed between the two ecological conditions (M and NM) on D30 and D90, with lower zooplankton density in mesocosms without macrophytes (*p* = 0.05 and *p* < 0.05, respectively; **Tables S9 and S10**). Significant differences were also found for the Shannon index between the two ecological conditions on D-7 and D90, being higher in the mesocosms with macrophytes (*p* < 0.01 and *p* < 0.05, respectively). Moreover, a significant reduction in zooplankton abundance was found for the highest difenoconazole concentration on D30 and D90 in the mesocosms with macrophytes, and on D30, D60 and D90 for the mesocosms without macrophytes. Species richness showed an increase in the mesocosm exposed to the highest difenoconazole concentration on D30 for the treatment with macrophytes (*p* < 0.05; **Tables S9-S11**); however, such differences were also present before the fungicide application on D-7.

**Fig. 2:**
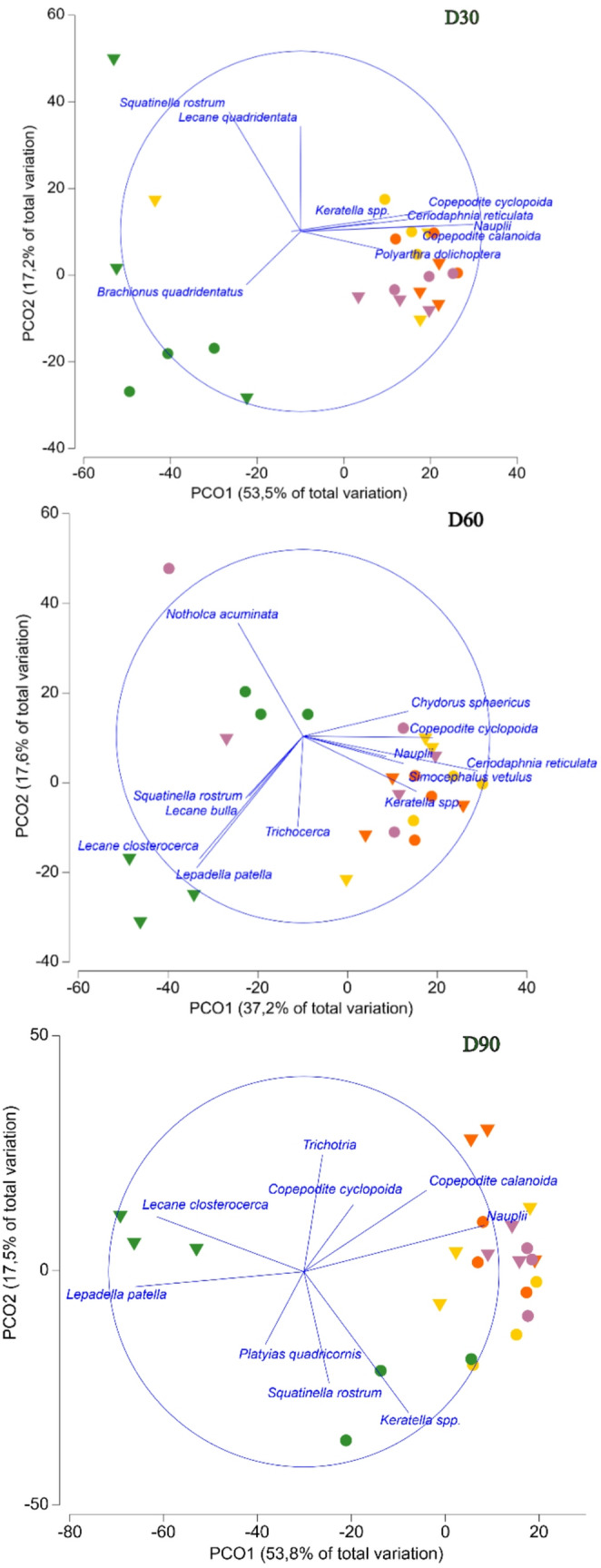
Principal Coordinates Analysis (PCoA) for zooplankton sampled on D30 (**A**), D60 (**B**) and D90 (**C**). Each point represents a mesocosm, the concentration of pollutant is represented by colours (■ 0; ■ 2; ■ 20; ■ 200 µg/L) and the two ecological conditions are represented by different symbols (●NM; ▼M)

The PERMANOVA showed significant differences in the zooplankton community between the M and NM condition (Table 1). Species of the genus *Keratella* (comprising the species *K. tropica*, *K. quadrata* and *K. cochlearis*) were more abundant in mesocosms without macrophytes (NM), whereas other rotifer genera, such as *Lecane*, and cladocerans, such as *Chydorus sphaericus* and *Simocephalus vetulus*, were more abundant in mesocosms containing macrophytes (M). The statistical test also revealed significant differences in community composition related to the fungicide exposure in all the sampling dates. The pair-wise test for mesocosms without macrophytes showed differences on D-7 between mesocosms with 2 µg/L and mesocosms with 20 and 200 µg/L, but not with the control treatment. For the mesocosms with macrophytes, differences on D-7 were also observed between the control and the 2 and 20 µg/L treatments, but not with 200 µg/L treatment. These did not show a treatment-related response, so were considered random effects that did not necessarily influence the effects observed after the application of the tested compound due to the time span between samplings and the differences on the kind of structural effects between both samplings (**Figure ****S2**). On D30 and D60 a significant effect was observed for the highest fungicide concentration (200 µg/L) in the mesocosms without macrophytes, and on D60 and D90 for the mesocosms with macrophytes, which differed from the other three treatments (*p* < 0.01 for both conditions). A significant interaction was identified between macrophyte condition (M and NM) and fungicide exposure on D90 (*p* < 0.01), with significant differences being found in mesocosms with macrophytes for the treatment with the highest concentration of difenoconazole (NOEC = 20 µg/L), while no significant differences were observed in mesocosms without macrophytes. The groupings of the zooplankton community assemblages from D30, D60 and D90 are shown in the PCoA plots (Fig. 2). *Keratella* spp. and copepodites were the taxa most affected by the fungicide at the highest test concentration.

**Table 1:** Results of the PERMANOVA (p-values shown) for the zooplankton and the macroinvertebrate communities. The table shows the effect of the two ecological conditions (Macrophytes), the effect of the fungicide concentrations (Difenoconazole), and their interaction (Interaction). D: day relative to the first Difenoconazole application. * p-value < 0.05; ** p-value < 0.01. On D60, the macroinvertebrate community was not sampled

Taxonomic group	Factors	D-7	D30	D60	D90
Zooplankton	Macrophytes	0.018*	0.389	0.014*	0.001**
	Difenoconazole	0.003**	0.001**	0.002**	0.001**
	Interaction	0.210	0.446	0.058	0.006**
Macroinvertebrates	Macrophytes	0.002**	0.014*	-	0.001**
	Difenoconazole	0.333	0.001**	-	0.001**
	Interaction	0.953	0.827	-	0.145

**Fig. 3:**
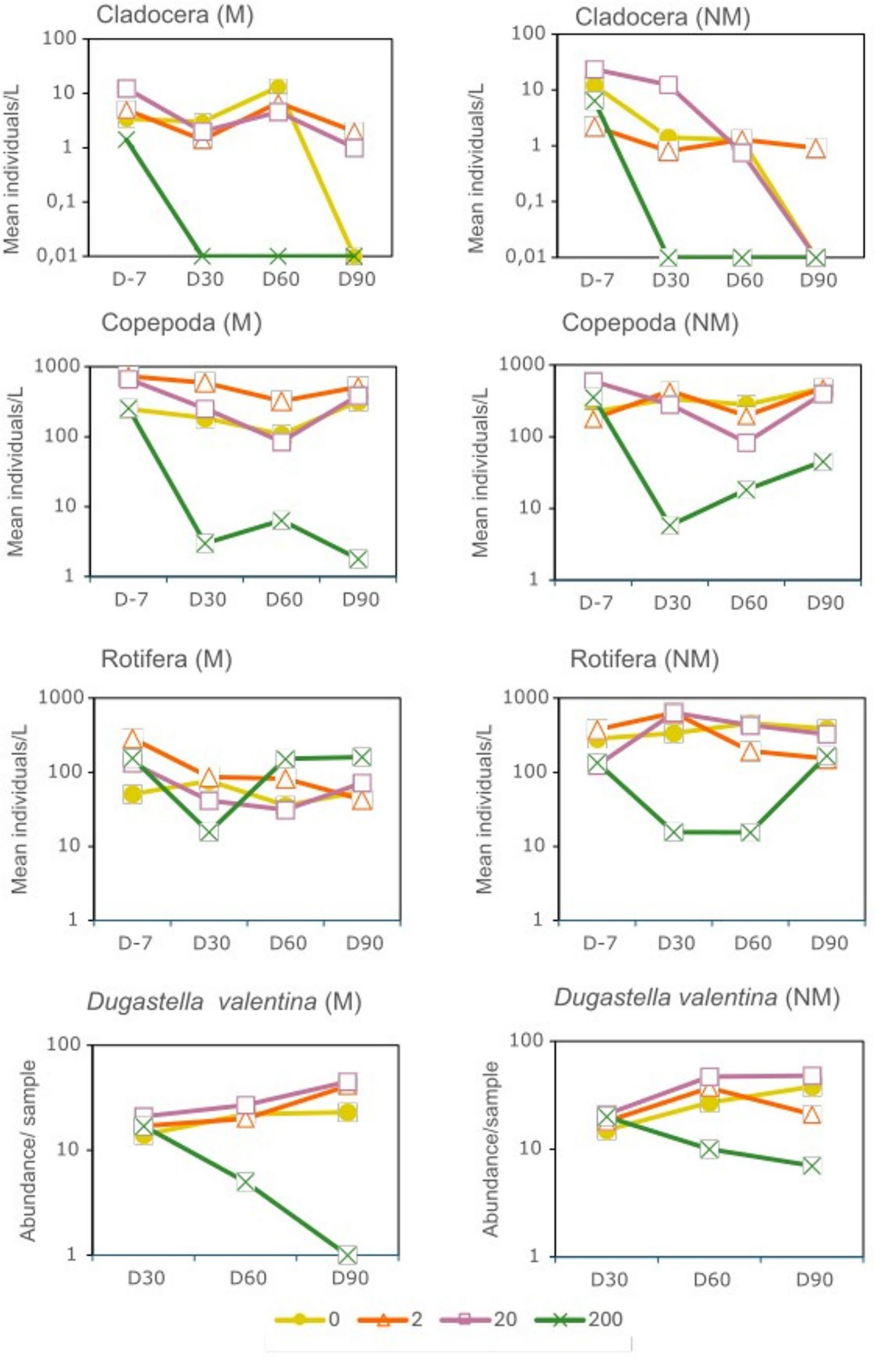
Abundance of the main groups of zooplankton (expressed as individuals/L) and most responding macroinvertebrate taxon, *Dugastella valentina*, (expressed as individuals/sample) in the mesocosms exposed to 0, 2, 20 and 200 µg/L of difenoconazole under different ecological conditions (M, with macrophytes; NM, without macrophytes). The sampling days (D) refer to days relative to the first fungicide application.

The William´s test for the principal groups of zooplankton revealed significant differences related to the difenoconazole concentrations showing a decrease in abundance of the three zooplankton groups (Table 2). In the macrophytes treatment, a decline in the abundance of cladocerans was observed both on D30 and D60 in the highest tested concentration (NOEC = 20 µg/L in both cases), but recovery was observed by day 90 (NOEC > 200 µg/L). For the mesocosms without macrophytes, a decline in the cladoceran abundance was observed on D60 (NOEC = 2 µg/L), but no significant effects of difenoconazole were found on D30. The abundance of copepods declined in the highest test concentration in the treatment with macrophytes on D60 and D90 (NOEC = 20 µg/L). Similarly, a reduction in copepod abundance was observed in the mesocosms without macrophytes on D30, D60 and D90 (NOEC = 20 µg/L in all three samplings). Within the copepod group, a greater effect was observed for cyclopoids on D60 for both ecological conditions (NOEC = 2 µg/L for M treatment and NOEC < 2 µg/L for the NM treatment). The abundance of copepod nauplii was reduced at the highest test concentration on D60 and D90 in mesocosms with macrophytes (NOEC = 20 µg/L), and on D30, D60 and D90 in the mesocosms without macrophytes (NOEC = 20 µg/L). Regarding rotifers, a decline in their abundance was noted on D30 in the mesocosms with macrophytes for the highest difenoconazole concentration (NOEC = 20 µg/L). Additionally, a reduction in their abundance was observed on D30 and D60 in the mesocosms without macrophytes (NOEC = 20 µg/L for both days). Within rotifers, the most responding taxa were *Polyarthra dolichoptera*, which was affected by all tested concentrations in the mesocosms without macrophytes (NOEC < 2 µg/L) on D60, and *Keratella* spp., which was affected by the highest tested concentration (NOEC = 20 µg/L) in mesocosms representing both ecological conditions on D30 and D60 (Table 2) (Fig. 3). An increase in the rotifer group *Lecane* spp. was also observed on D60 and D90 in the mesocosms with macrophytes (Table 2).

**Table 2:** Calculated NOECs (No Observed Effect Concentrations) for the zooplankton and macroinvertebrate communities and for the different taxa. Only main zooplankton groups and the taxa that showed significant effects in at least one sampling day are displayed. ↑: abundance increase; ↓: abundance decrease. D: day relative to the first Difenoconazole application. M: macrophytes treatment; NM: Non-macrophytes treatment; n.a.: not assessed; noecs are expressed in µg/L. The super-index refers to the exclusion criterion: ^a^ there was insufficient abundance of the taxon; ^b^ effects did not occur in a dose-response manner. - taxon not present, so responses could not be evaluated

	M				NM			
	D-7	D30	D60	D90	D-7	D30	D60	D90
Zooplankton								
**Community**	> 200	> 200	20	20	> 200	20	20	> 200
**Cladocera**	> 200	20↓	20↓	> 200^a^	> 200	> 200^a^	2↓^a^	> 200^a^
**Copepoda**	> 200	> 200	20↓	20↓	> 200	20↓	20↓	20↓
Copepodite Cyclopoida	> 200	> 200	2↓	> 200	20↓	> 200	< 2↓	> 200
Copepodite Calanoida	> 200	> 200	> 200	20↓	2↑	> 200	> 200	> 200
Nauplii	> 200	> 200	20↓	20↓	> 200	20↓	20↓	20↓
**Rotifera**	> 200	20↓	> 200	> 200	> 200	20↓	20↓	> 200
*Brachionus calyciflorus*	< 2↓^a^	-	-	-	> 200	> 200	-	-
*Brachionus havanaensis*	> 200	> 200	-	-	< 2↓^a^	> 200	> 200	-
*Platyias quadricornis*				> 200		> 200		20↑^a^
*Testudinella* sp	> 200	> 200	2↓^a^	> 200	> 200	> 200	> 200	
*Hexarthra* sp						> 200	> 200	20↓^a^
*Polyarthra dolichoptera*	> 200	> 200	> 200	> 200	> 200	> 200	< 2↓	> 200
*Keratella* spp.	> 200	20↓	> 200	20↓	> 200	20↓	20↓	> 200
*Lecane* spp.	> 200	> 200	20↑	20↑	> 200	> 200	> 200	> 200
**Macroinvertebrate**								
**Community**	> 200	> 200	n.a.	20↓	> 200	> 200	n.a.	> 200
*Dugastella valentina*	> 200	20↓	n.a.	20↓	> 200	> 200	n.a.	20↓
*Palaemonetes zariquieyi*	> 200	> 200	n.a.	20↓	> 200	> 200	n.a.	> 200
*Cloeon* sp.	> 200	> 200	n.a.	> 200	> 200	> 200	n.a.	< 2↓^a^
Chironomidae	> 200	< 2↑^b^	n.a.	> 200	20↑^b^	> 200	n.a.	> 200
*Orthochladiinae* sp.	> 200	> 200	n.a.	> 200	> 200	-	n.a.	20↑^a^
*Gerris* sp.	-	> 200	n.a.	> 200	< 2↓^a^	-	n.a.	-
*Notonecta* sp.	> 200	20↑	n.a.	> 200	> 200	20↑	n.a.	> 200
*Hydroglyphus* sp.	> 200	20↓^b^	n.a.	> 200	> 200	> 200	n.a.	> 200
*Melanopsis* sp.	> 200	> 200	n.a.	> 200	20↑^a^	20↑^a^	n.a.	> 200
Planorbidae	> 200	20↓	n.a.	2↓	> 200	> 200	n.a.	> 200

### Macroinvertebrate community

During the experimental period, 25 macroinvertebrate taxa were identified, most of them belonging to Insecta (16 taxa), followed by Crustacea (4), Mollusca (3), Platyhelminthes (1) and Araneae (1). The most abundant group was the family Chironomidae (Diptera), followed by the snail species *Physella acuta*, the family Planorbidae and the Decapoda *Dugastella valentina*.

Significant differences were identified in the abundance of macroinvertebrates between the two ecological conditions, with a higher total abundance in the mesocosms with macrophytes on D30 and D90 (*p* < 0.05). Additionally, a higher abundance was observed for the highest difenoconazole concentration in the mesocosms without macrophytes on D-7. Furthermore, a reduction in the Shannon diversity index was detected for the highest fungicide concentration in the macrophyte treatment on D30 (**Tables S9-S11**).

**Fig. 4:**
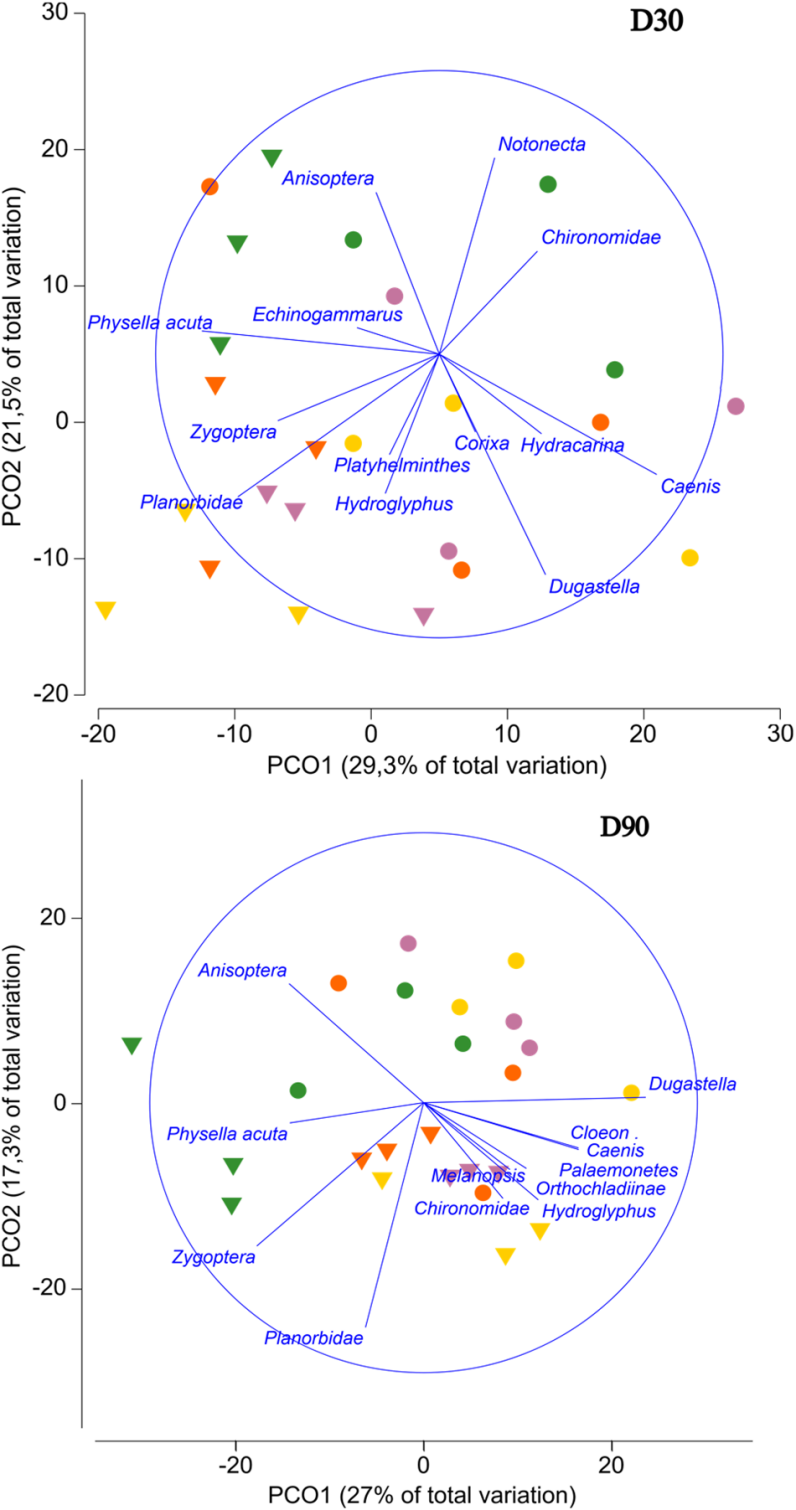
Principal Coordinates Analysis (PCoA) for macroinvertebrate taxa on D30 and D90. Each point represents a mesocosm, the concentration of difenoconazole (µg/L) is shown by colors (■ C; ■ 2; ■ 20; ■ 200 µg/L) and the two ecological conditions are represented by different symbols (●NM; ▼M). Distances between points are proportional to similarities in community composition

The PERMANOVA showed a significant effect between the two ecological conditions (M and NM) for all the sampling dates (*p* < 0.05). Additionally, significant effects of the fungicide were found on D30 and D90 (Table 1). The differences between the macroinvertebrate community assemblages sampled on D30 and D90 can be visualized in the PCoA plots (Fig. 4).

The William´s test showed a decrease in the abundance of *D. valentina* at the highest fungicide concentration on D30 and D90 (NOEC = 20 µg/L) for the macrophytes treatment, while in the mesocosms without macrophytes, *D. valentina* showed a reduction in their abundance only on D90 (NOEC = 20 µg/L) (Fig. 3). The decapod *Palaemonetes zariquieyi* exhibited a decline in its population on D90 in the macrophyte treatment for the highest concentration of difenoconazole (NOEC = 20 µg/L). In the presence of macrophytes, the abundance of the snail family Planorbidae decreased, with NOEC values of 20 µg/L on Day 30 and 2 µg/L on Day 90. This effect was not observed in macrophyte-free mesocosms, where Planorbidae abundance was consistently very low (Table 2). On D30, a decrease in the population of the coleopteran *Hydroglyphus* sp. was observed for the highest test concentration in the macrophyte treatment (NOEC = 20 µg/L). Conversely, *Notonecta* sp. exhibited an increase in abundance on D30 in both ecological conditions in mesocosms exposed to the highest concentration of difenoconazole (NOEC = 20 µg/L).

## Discussion

This study provides the first assessment of difenoconazole exposure dynamics and toxic effects on aquatic populations and communities representative of the Mediterranean region. Difenoconazole exhibited dissipation times (DT50s) that are within the range shown by the experiments by Bromilow et al. (2003, 2006) for outdoor mesocosms without macrophytes under temperate conditions. Our study shows that difenoconazole affected aquatic communities differently under the two tested ecological conditions (i.e., with and without macrophytes). The differential sensitivity observed between the two treatments is likely attributable to faster difenoconazole dissipation in the vegetated mesocosms. Macrophytes have been shown to reduce chemical bioavailability for pelagic organisms by absorption of chemicals (Stang et al. 2016), and to affect the structure of microbial communities, potentially favouring microbial assemblages that are more prone to the biodegradation of organic contaminants (Rodrigo et al. 2022).

Furthermore, the differences in the effects produced by difenoconazole in the two ecological conditions can be attributed to the differential species composition of each of the treatments. In the treatment with macrophytes, rotifer groups such as *Lecane* spp., cladocerans such as *Chydorus sphaericus*, and gastropods such as Planorbidae were more abundant, whereas in the treatment without macrophytes, pelagic zooplankton groups associated with the water column, such as *Keratella* spp. and the calanoid copepod *Copidodiaptomus numidicus* dominated. Such differences in species composition may be related to the different physico-chemical and biotic conditions created by the macrophytes in the treatments and habitat preferences of these species. The presence of macrophytes modifies the habitat, increasing the surface for periphyton and promotes higher concentrations of dissolved oxygen and elevated pH, resulting from increased photosynthetic activity and greater carbon dioxide uptake. This modified environment is known to support larger populations of Cladocera and other macrozooplankton taxa, as observed in previous studies (Grillo-Avila et al. 2024; Roessink et al. 2005).

The results of this experiment demonstrate that difenoconazole had no significant effect on the decomposition of leaf-litter organic matter. These results are in line with those obtained in previous experiments, where it was observed that exposure to triazole fungicides does not affect organic matter decomposition (Pimentão et al. 2020; Zubrod et al. 2015). Some studies suggest that although the fungal community structure can be affected by difenoconazole, such effects do not affect total fungal biomass nor the leaf decomposition process (Dimitrov et al. 2014), suggesting a high level of functional redundancy within the microbial community (Pascoal et al. 2005; Pimentão et al. 2020).

In our experiment, the zooplankton community showed a higher sensitivity to difenoconazole when compared to the macroinvertebrate community. The study shows a zooplankton community NOEC of 20 µg/L for both ecological conditions; however, different responses were observed among zooplankton taxonomic groups and species. Cladocerans showed a high sensitivity to difenoconazole. A NOEC of 20 µg/L was calculated for Cladocera in the mesocosms with macrophytes, while in the mesocosms without macrophytes the lowest NOEC was 2 µg/L in one isolated sampling day, although this last effect should be treated with caution due to the low cladoceran density in the controls. A clear effect was also observed at the highest concentration in the treatment without macrophytes (NOEC = 20 µg/L). These results are consistent with the chronic NOEC reported by Sun et al. (2024) for *Daphnia magna*, and previous experiments showing effects on development in daphnid juveniles (Mendieta-Herrera et al. 2023). Copepods also showed high sensitivity to difenoconazole, particularly cyclopoid copepodites, which showed a NOEC of 2 µg/L in the mesocosms without macrophytes, and < 2 µg/L in the mesocosms with macrophytes in one single sampling date, while significant nauplii declines were only recorded at the highest test concentration (NOEC = 20 µg/L). Rotifers were also found to be susceptible to elevated levels of difenoconazole, exhibiting a consistent decline in the mesocosms without macrophytes (effects observed on D30 and D60). This response was particularly evident for *P. dolichoptera* and *Keratella* spp., two planktonic rotifers commonly present in the water column.

The available literature on the impacts of difenoconazole on zooplankton is limited, therefore the findings of this experiment provide novel knowledge and suggest copepods and some rotifer taxa as having equal or greater sensitivity to difenoconazole compared to Cladocera. Other studies, such as Moreira et al. (2021), reported no significant impact of difenoconazole on zooplankton communities at concentrations of 3.6 µg/L and 6.6 µg/L, although the compound was applied with runoff water, a factor that may have lowered its bioavailability through sorption to suspended particles.

In general, an abundance recovery of rotifers and copepods was observed at the end of the experiment (D90), while no such recovery was observed for cladocerans. Such differences in internal recovery capacity are most likely related to the different reproductive traits among these species (Bundschuh et al. 2023). The decline in Cladocera may, in turn, have favoured the proliferation of other filter-feeding organisms, such as Rotifera (Polla et al. 2022; Rico-Martinez et al. 2012; Zhao et al. 2021). This is exemplified for *Lecane* spp., which showed a population increase during the recovery period following the administration of the compound, mainly in the mesocosm that contained macrophytes.

The observed slight increase in phytoplankton biomass (based on the chlorophyll-a concentration) on D90 under the highest treatment concentration may be attributable to the fungicide’s impact on zooplankton. The zooplankton community was dominated by phytoplankton-filtering taxa, particularly efficient grazers such as cladocerans and calanoids. This interpretation is supported by previous studies, which have shown that a reduction in grazing pressure due to organic contaminants can trigger a phytoplankton increase comparable to that seen in moderately eutrophic ecosystems (Cottingham et al. 2004; Fu et al. 2021).

The most significant impact of difenoconazole on macroinvertebrate communities was observed in the mesocosms with macrophytes on the last sampling day (NOEC = 20 µg/L). In contrast, no significant effects were observed at the community level in the mesocosms without macrophytes (NOEC > 200 µg/L). The taxon that demonstrated the greatest sensitivity to the fungicide action was the snail family Planorbidae (NOEC = 2 µg/L) in the macrophytes treatment at the end of the experiment. The calculated NOEC for this taxon was significantly lower than the chronic toxicity thresholds reported in the literature for other molluscs, such as *Crassostrea virginica* (NOEC = 180 µg/L; EPA 1992).

The macrocrustacean *Dugastella valentina*, which is an endangered decapod in many Mediterranean wetlands, was also significantly affected. *D. valentina* exhibited a decline in abundance in both treatments at the highest test concentration resulting in a NOEC of 20 µg/L. Previous studies show that crustacea is a sensitive group to difenoconazole, with the lowest chronic NOEC reported as 48 µg/L for the Mysida *Americamysis bahia* (EPA 1992). Furthermore, evidence has demonstrated that azole fungicides can exert direct and indirect effect on detritivorous organisms, resulting from the impact of the fungicide on their primary food resources (Bundschuh et al. 2011; Zubrod et al. 2014).

The sensitivity of some aquatic insects to difenoconazole was also evident, notably in the coleopteran larvae of *Hydroglyphus* sp., whose abundance declined at the highest test concentration. This result aligns with the findings of Amador et al. (2024), who reported indirect effects of the fungicide azoxystrobin on Coleoptera predators in Mediterranean wetlands. Specifically, they proposed that declines in predatory insect populations could be driven by fungicide-induced reductions in prey, such as filter feeders and herbivorous invertebrates. In our study, the population decline of the small coleopteran *Hydroglyphus* sp. may similarly be explained by a reduction in zooplankton abundance, which constitutes a primary food source for this genus (Millán et al. 2014).

Our study shows that community-level effects of difenoconazole on zooplankton and macroinvertebrates may only occur at concentrations above 20 µg/L, although some taxa may show significant population declines at concentrations of 2 µg/L in isolated sampling days (i.e., Cyclopoid copepods). Therefore, the chronic HC5 value calculated by Sun et al. (2024) based on laboratory toxicity data for primary producers, invertebrates and fish (0.96 µg/L) may be sufficiently protective for zooplankton and macroinvertebrate communities, but an assessment factor to the calculated HC5 may be recommended to ensure sufficient protection of aquatic populations under field conditions. In accordance with the EFSA guidance for evaluating plant protection products in freshwater ecosystems (EFSA, 2013), an assessment factor of at least 3 is recommended. This recommendation was supported by Rico et al. (2019), who compared regulatory acceptable concentrations from chronic SSDs for fungicides with NOECs from micro- and mesocosm experiments. They concluded that such a factor is often necessary for fungicides with a biocidal mode of action to adequately protect freshwater populations and communities under (semi-)field conditions.

Our study indicates that peak exposure concentrations of difenoconazole, estimated at 10 µg/L in rice fields and 1–5 µg/L in drainage ditches (Amador et al. 2025), may cause short-term population-level effects on zooplankton (e.g., Cyclopoid copepods) and macroinvertebrate taxa (e.g., Planorbidae). Zooplankton serve as key ecosystem grazers of phytoplankton and as a primary food source for fish and other vertebrates (Anton-Pardo and Armengol 2014; Hanazato 1998), effectively constituting a keystone group in Mediterranean coastal wetlands. In these often-eutrophic systems, a reduction in zooplankton grazing pressure can lead to increased phytoplankton biomass and the proliferation of potentially toxic cyanobacterial blooms (Ger et al. 2016). Likewise, aquatic macroinvertebrates, such as freshwater snails, represent an important food resource for birds and other aquatic and terrestrial fauna in Mediterranean wetlands (Covich et al. 1999; Jadhav et al. 2022).

In summary, this study provides a detailed analysis of the potential impacts of the fungicide difenoconazole on aquatic communities representative of Mediterranean wetlands under differing macrophyte conditions. It highlights the mitigating role of macrophytes in reducing difenoconazole exposure and reveals a high sensitivity of certain zooplankton taxa under prolonged exposure, with slight variations depending on macrophyte presence. Overall, the findings suggest that zooplankton and macroinvertebrate communities are likely unaffected at environmentally relevant concentrations found in rice paddies and adjacent freshwater ecosystems, although short-term population declines of copepods and some snail taxa may occur. The ecological risks associated with azole fungicides are further compounded by their potential to induce additive or synergistic effects when combined with other pesticides, such as the fungicide azoxystrobin (Fu et al. 2018; Gottardi and Cedergreen 2019; Rösch et al. 2017). This is particularly significant given that difenoconazole is commonly applied in combination with azoxystrobin to prevent and treat fungal infections in rice fields (Amador et al. 2025; Martínez-Megías et al. 2023). Further experimental research is needed to assess the potential additive or synergistic toxic effects of pesticide mixtures containing difenoconazole and other compounds used in rice cultivation, and to develop effective risk mitigation measures for downstream aquatic ecosystems.

## Supplementary Information

Below is the link to the electronic supplementary material.

Supplementary Material 1

Supplementary Material 2

## Data Availability

data is provided within the manuscript or supplementary information files.

## Declarations

### Data statement

The raw data of this experiment is provided in the Supporting Information file II.
