# Supplementary material for: Responses of mediterranean freshwater invertebrates to the fungicide difenoconazole across different macrophyte dominance conditions: A mesocosm study
Source: Ecotoxicology. 2026 Feb 18;35(3):58. doi: 10.1007/s10646-026-03042-7 (PMC12917022; doi:10.1007/s10646-026-03042-7)
Supplement: Supplementary file 1 — Supplementary Material 1 [file 10646_2026_3042_MOESM1_ESM.docx]

**Supporting information I**

**Text S1. Analytical method**

**Standards and reagents used in the analytical method**

The analytical standard of difenoconazole (purity ≥ 95%) for the quantitative analysis was purchased from Merck (Darmstadt, Germany). LC/MS-grade acetonitrile and methanol were supplied from Scharlau (Barcelona, Spain). Formic acid (purity ≥98%) was obtained from Merck. High purity water was obtained from a Milli-Q water purification system (Millipore, Mildford, MA, USA). Stock solutions of difenoconazole for the analytical measurements were prepared at the concentration level of 2000 mg L^-1^ in MeOH and stored in amber glass vials at -20 °C. The working solutions at 1 mg L^-1^ in MeOH and calibration solutions (0.5, 1, 3, 5, 10, 20 and 30 µg/L) in MeOH: water (10:90, v/v) were prepared daily.

**Quantification by LC-MS/MS**

The quantification of difenoconazole in samples was carried out using a liquid chromatograph (LC) (1200 series, Agilent Technologies, USA) coupled to a triple quadrupole (MS/MS) mass spectrometer (6495A, Agilent Technologies), equipped with an electrospray ionization (ESI) interface and Agilent Jet Stream Technology. Ions were generated using an electrospray ion source in negative mode. Analyses were done in Multiple Reaction Monitoring (MRM) mode recording three precursor/product ion transitions (quantifier (Q) and qualifier (q1)). Selected ions, together with their corresponding CE and abundance percentages are listed in **Table S1**. The column used was an ACE C18 (50 x 2.1 mm x 3 µm), supplied by Symta. Mobile Phase A was ultrapure water containing 0.1% of formic acid. Phase B was acetonitrile containing 0.1% of formic acid. A 19 min gradient started at 5% B to 100% B solvent, then maintained for 1 min. A 4 min post-run time was used after each analysis. The injection volume, the column temperature and flow rate were 20 μL, 40°C and 0.6 mL/min. Samples were centrifuged (13000 rpm, 5 min) and diluted (1/10) according to instrumental requirements for the LC-MS/MS analysis.

The Limit of Quantification (LOQ) was 0.5 µg/L. It was determined as the lowest concentration whose quantification transition presented a signal-to-noise ratio (S/N) = 10, and qualification transition was detected accomplishing abundance criteria. The Limit of Detection (LOD) was 0.05 µg/L. It was determined as the minimum detectable amount of analyte with a signal-to-noise ratio (S/N) = 3, maintaining abundance criteria between transitions.

**Table S1.** MRM Method Parameters. RT: retention time.

| **Compound** | **RT (min)** | **Precursor ion** | **Product ion** | **Transition** | **Collision energy (V)** | **Abundance (%)** |
| --- | --- | --- | --- | --- | --- | --- |
| difenoconazole | 14.7 | 405.9 | 250.8 | Q | 16 |  |
|  |  |  | 336.8 | q1 | 28 | 20 |

**Table S2.** Measured concentrations of difenoconazole (mean ± standard deviation, µg/L) in the different treatments (NM: non-macrophytes; M: macrophytes). (Before) and (after) indicate whether the sample was taken before or after the chemical application.

|  | **NM** | | | **M** | | |
| --- | --- | --- | --- | --- | --- | --- |
| **Sampling day** | **2 µg/L** | **20 µg/L** | **200 µg/L** | **2 µg/L** | **20 µg/L** | **200 µg/L** |
| **D0** | 2.5±0.7 | 16±3.2 | 198±15 | 2.1±0.1 | 18±1.6 | 179±6.5 |
| **D7** | 1.5±0.1 | 15±2.2 | 151±4.4 | 1.5±0.2 | 13±3.4 | 150±6.2 |
| **D14 (before)** | 1.0±0.1 | 13±1.4 | 134±5.8 | 1.0±0.1 | 14±0.7 | 138±3.6 |
| **D14 (after)** | 3.2±0.8 | 42±9.5 | 334±29 | 4.1±0.8 | 50±12 | 439±68 |
| **D21** | 2.4±0.3 | 24±2.6 | 302±14 | 2.3±0.1 | 24±0.6 | 296±23 |
| **D30** | 2.0±0.3 | 26±0.7 | 293±32 | 1.9±0.1 | 28±1.1 | 292±26 |
| **D45** | 1.5±0.3 | 19±1.4 | 240±25 | 1.3±0.2 | 24±0.8 | 245±26 |

**Table S3.** Calculated dissipation coefficient (*k*) and half-life (DT50) of difenoconazole in the different treatments. NM: non-macrophytes; M: macrophytes.

|  | **NM** | | | **M** | | |
| --- | --- | --- | --- | --- | --- | --- |
|  | **2 µg/L** | **20 µg/L** | **200 µg/L** | **2 µg/L** | **20 µg/L** | **200 µg/L** |
| ***k* (d^-1^)** | 0.025 | 0.021 | 0.010 | 0.033 | 0.018 | 0.016 |
| **DT50 (d)** | 28 | 33 | 68 | 21 | 39 | 43 |
| **Mean DT50 (d)** | 43 | | | 34 | | |

**Table S4**. Average values of water temperature, conductivity, pH, Oxygen (O_2_), concentration of soluble reactive phosphorus (SRP), Total phosphorus (TP), nitrate (NO_3_), ammonium (NH_4_), Dissolved Inorganic Nitrogen (DIN), total suspended solids (TSS), chlorophyl-a (Chl-a) and alkalinity in each treatment. M: Macrophytes; NM: Non-macrophytes; Con: difenoconazole concentration.

|  |  | **Conc. (µg/L)** | **Water temperature (Cº)** | **Conductivity (µS/cm)** | **pH** | **O2 (mg/l)** | **SRP (µM)** | **P (µM)** | **NO_3_^-^ (µM)** | **NH_4_ (µM)** | **DIN (µM)** | **TSS (mg/l)** | **Chl-a (µg/l)** | **Alkalinity (meq/L)** |
| --- | --- | --- | --- | --- | --- | --- | --- | --- | --- | --- | --- | --- | --- | --- |
| D-7 | M | 0 | 17.53±0.4 | 1112.67±11.44 | 9.21±0.09 | 9.33±0.67 | 0.13±0.04 | 0.3±0.11 | 23.69±0.96 | 1.76±0.55 | 25.44±1.11 | 0.95±0.25 | 2.14±0.1 | 1.67±0.02 |
|  |  | 2 | 17.5±0.29 | 1057.67±14.82 | 9.27±0.05 | 10.52±0.37 | 0.09±0.03 | 0.26±0.05 | 21.93±2.63 | 1.76±0.27 | 23.68±2.54 | 0.62±0.19 | 1.83±0.45 | 1.36±0.14 |
|  |  | 20 | 17.93±0.45 | 1085±35.81 | 9.28±0.04 | 10.13±0.42 | 0.22±0.11 | 2.57±3.04 | 24.85±1.71 | 1.45±0.12 | 26.3±1.58 | 1.57±0.33 | 1.78±0.58 | 1.63±0.1 |
|  |  | 200 | 18.33±0.45 | 1101±34.71 | 9.18±0.02 | 9.83±0.18 | 0.16±0.03 | 0.63±0.22 | 24.71±1.48 | 2.77±0.98 | 27.49±2.46 | 1.3±0.24 | 1.68±0.21 | 1.83±0.19 |
|  | NM | 0 | 17.9±0.57 | 1129.67±31.9 | 9.1±0.07 | 10.73±1.09 | 0.14±0.07 | 0.73±0.37 | 26.1±3.09 | 1.49±0.44 | 27.59±3.33 | 1.98±0.76 | 3.07±1.14 | 1.61±0.13 |
|  |  | 2 | 18±0.57 | 1099±14.35 | 8.98±0.05 | 9.11±0.23 | 0.11±0.05 | 2.09±0.58 | 24.58±2.21 | 2.71±1.03 | 27.3±3.22 | 2.65±0.63 | 3.46±0.66 | 1.73±0.48 |
|  |  | 20 | 18.17±0.84 | 1110.33±25.9 | 9.15±0.12 | 10.57±1.08 | 0.13±0.04 | 1.8±1.1 | 25.09±2.51 | 1.95±0.31 | 27.04±2.8 | 2.01±0.91 | 3.29±0.8 | 1.68±0.37 |
|  |  | 200 | 17.37±0.26 | 1104±36.77 | 9.01±0.05 | 9.15±0.43 | 0.16±0.03 | 0.63±0.31 | 25.43±2.11 | 1.35±0.42 | 26.79±2.4 | 2.86±1.34 | 4.94±2.9 | 1.79±0.13 |
| D30 | M | 0 | 12.07±0.05 | 1201±12.83 | 9.43±0.1 | 12.75±0.76 | 0.37±0.09 | 0.44±0.08 | 27.99±2.48 | 0.79±0.19 | 28.78±2.62 | 0.55±0.18 | 1.58±0.58 | 1.29±0.08 |
|  |  | 2 | 11.87±0.05 | 1162.67±5.44 | 9.35±0.2 | 12.05±0.69 | 0.48±0.07 | 0.46±0.15 | 24.5±2.93 | 0.42±0.15 | 24.92±2.93 | 0.27±0.09 | 0.94±0.26 | 1.31±0.29 |
|  |  | 20 | 12.23±0.63 | 1165.67±42.68 | 9.43±0.09 | 12.36±0.65 | 0.33±0.04 | 0.55±0.14 | 22.63±2.03 | 0.19±0.05 | 22.82±1.98 | 0.71±0.06 | 1.23±0.31 | 1.24±0.15 |
|  |  | 200 | 13.07±0.83 | 1183.33±20.29 | 9.29±0.18 | 11.95±1.04 | 0.55±0.12 | 0.92±0.52 | 28.39±1.9 | 0.61±0.05 | 29±1.86 | 0.75±0.39 | 1.03±0.43 | 1.25±0.19 |
|  | NM | 0 | 12.27±0.33 | 1234±44.92 | 8.7±0.47 | 11.27±0.59 | 0.42±0.26 | 0.71±0.18 | 26.48±3.35 | 1.48±0.87 | 27.96±3.4 | 1.99±0.68 | 2.49±1.3 | 1.61±0.37 |
|  |  | 2 | 12.57±0.78 | 1219.67±18.12 | 9.08±0.24 | 11.59±0.4 | 0.35±0.11 | 0.61±0.13 | 25.32±1.72 | 0.52±0.34 | 25.84±2.06 | 1.54±0.53 | 1.44±0.2 | 1.75±0.08 |
|  |  | 20 | 12.37±0.39 | 1214±41.02 | 9.04±0.1 | 11.35±0.15 | 0.48±0.07 | 1.02±0.38 | 25.99±1.82 | 1.05±0.66 | 27.05±2.04 | 1.61±0.07 | 1.3±0.51 | 1.49±0.33 |
|  |  | 200 | 11.93±0.34 | 1227±34.29 | 8.77±0.07 | 10.75±0.53 | 0.48±0.09 | 0.63±0.36 | 32.19±1.78 | 0.38±0.2 | 32.57±1.62 | 1.07±0.56 | 1.47±0.9 | 1.95±0.25 |
| D90 | M | 0 | 12.23±0.17 | 1254±19.8 | 8.59±0.23 | 12.45±0.7 | 0.18±0.05 | 0.3±0.1 | 28.46±2.44 | 3.2±0.83 | 31.66±2.16 | 0.75±0.43 | 0.74±0.01 | 0.79±0.1 |
|  |  | 2 | 12.23±0.05 | 1247.67±6.55 | 8.51±0.19 | 12.53±0.57 | 0.13±0.04 | 0.2±0 | 27.93±0.23 | 2.99±0.94 | 30.92±1.11 | 0.56±0.14 | 1.24±0.41 | 1.16±0.25 |
|  |  | 20 | 12.13±0.17 | 1218.33±38.58 | 8.62±0.34 | 12.33±0.28 | 0.29±0 | 0.53±0.19 | 26.38±0.85 | 7.7±2.42 | 34.09±1.76 | 0.51±0.22 | 0.78±0.1 | 0.8±0.03 |
|  |  | 200 | 12.1±0.14 | 1237±15.58 | 8.77±0.4 | 12.52±0.57 | 0.21±0.13 | 0.53±0.19 | 31.93±1.8 | 7.53±7.07 | 39.46±5.39 | 0.54±0.13 | 2.85±1.09 | 1.19±0.32 |
|  | NM | 0 | 12.13±0.09 | 1313.33±33.64 | 8.28±0.07 | 11.7±0.48 | 0.2±0.11 | 0.55±0.17 | 28.11±3.14 | 7.51±7.42 | 35.62±8.81 | 1.26±0.19 | 1.26±0.25 | 1.45±0.25 |
|  |  | 2 | 12.07±0.12 | 1272±47.46 | 8.3±0.03 | 11.61±0.22 | 0.23±0 | 0.5±0.13 | 26.71±0.72 | 5.48±2.91 | 32.19±3.61 | 1.23±0.21 | 1.32±0.56 | 1.52±0.47 |
|  |  | 20 | 12.23±0.05 | 1282.67±20.74 | 8.43±0.3 | 11.96±0.71 | 0.18±0.04 | 0.55±0.05 | 28.75±3.19 | 6.59±5.3 | 35.35±2.29 | 1.66±0.3 | 1.13±0.57 | 1.35±0.17 |
|  |  | 200 | 12.13±0.12 | 1292.33±31.48 | 8.15±0.06 | 11.35±0.09 | 0.15±0.05 | 0.55±0.22 | 31.79±0.52 | 4.96±3.53 | 36.75±3.03 | 1.01±0.08 | 2.38±0.63 | 2.81±1.58 |

**Table S5**. Results of ANOVA (p-values) for water temperature, conductivity, pH, dissolved oxygen, soluble reactive phosphorus (SRP), total phosphorus (TP), nitrate, ammonium, chlorophyll-a, alkalinity, total suspended solids (TSS), and organic matter (OM) decomposition. Macrophytes indicate the effect of the two ecological conditions. Difenoconazole indicates the effect of the chemical concentrations. and the Interaction indicates the interaction of both. The asterisks indicate statistically significant effects (p-value < 0.05). D: day relative to the first difenoconazole application.

|  |  | **D-7** | **D30** | **D90** |
| --- | --- | --- | --- | --- |
| **Water temperature** | **Macrophytes** | 0.898 | 0.923 | 0.597 |
|  | **Difenoconazole** | 0.792 | 0.804 | 0.848 |
|  | **Interaction** | 0.196 | 0.113 | 0.431 |
| **Conductivity** | **Macrophytes** | 0.136 | 0.009** | 0.003** |
|  | **Difenoconazole** | 0.222 | 0.558 | 0.465 |
|  | **Interaction** | 0.798 | 0.956 | 0.773 |
| **pH** | **Macrophytes** | <0.001** | <0.001* | 0.015* |
|  | **Difenoconazole** | 0.118 | 0.458 | 0.921 |
|  | **Interaction** | 0.272 | 0.511 | 0.587 |
| **Dissolved oxygen** | **Macrophytes** | 0.846 | 0.006** | 0.006** |
|  | **Difenoconazole** | 0.328 | 0.528 | 0.944 |
|  | **Interaction** | 0.037* | 0.729 | 0.724 |
| **SRP** | **Macrophytes** | 0.637 | 1.00 | 0.832 |
|  | **Difenoconazole** | 0.301 | 0.504 | 0.867 |
|  | **Interaction** | 0.489 | 0.409 | 0.552 |
| **TP** | **Macrophytes** | 0.539 | 0.304 | 0.069 |
|  | **Difenoconazole** | 0.211 | 0.474 | 0.224 |
|  | **Interaction** | 0.48 | 0.314 | 0.435 |
| **Nitrate** | **Macrophytes** | 0.186 | 0.182 | 0.868 |
|  | **Difenoconazole** | 0.607 | 0.009** | 0.016* |
|  | **Interaction** | 0.82 | 0.369 | 0.612 |
| **Ammonium** | **Macrophytes** | 0.85 | 0.111 | 0.733 |
|  | **Difenoconazole** | 0.44 | 0.135 | 0.82 |
|  | **Interaction** | 0.062 | 0.261 | 0.688 |
| **Chlorophyll-a** | **Macrophytes** | 0.007* | 0.165 | 0.668 |
|  | **Difenoconazole** | 0.778 | 0.253 | 0.002** |
|  | **Interaction** | 0.564 | 0.842 | 0.622 |
| **Alkalinity** | **Macrophytes** | 0.499 | 0.003** | 0.018* |
|  | **Difenoconazole** | 0.512 | 0.574 | 0.159 |
|  | **Interaction** | 0.575 | 0.598 | 0.481 |
| **TSS** | **Macrophytes** | 0.002** | <0.001** | <0.001** |
|  | **Difenoconazole** | 0.647 | 0.482 | 0.305 |
|  | **Interaction** | 0.426 | 0.24 | 0.191 |
| **OM decomposition** | **Macrophytes** | - | 0.003* | 0.094 |
|  | **Difenoconazole** | - | 0.876 | 0.953 |
|  | **Interaction** | - | 0.717 | 0.088 |

**Table S6.** Calculated NOECs (No Observed Effect Concentrations) for physico-chemical parameters, chlorophyll-a and organic matter decomposition. ↑: value increase; ↓: value decrease. D: day relative to the first difenoconazole application. M: Macrophytes treatment; NM: Non-macrophytes treatment. NOECs are expressed in µ/L.

|  | **M** | | | **NM** | | |
| --- | --- | --- | --- | --- | --- | --- |
| **Parameter** | **D-7** | **D30** | **D90** | **D-7** | **D30** | **D90** |
| **Temperature (ºC)** | 200 | 200 | 200 | 200 | 200 | 200 |
| **Conductivity (µS/cm)** | 200 | 200 | 200 | 200 | 200 | 200 |
| **pH** | 200 | 200 | 200 | 200 | 200 | 200 |
| **O_2_ (ppm)** | 200 | 200 | 200 | 200 | 200 | 200 |
| **SRP (µM)** | 200 | 20↑ | 200 | 200 | 200 | 200 |
| **Total P (µM)** | 200 | 200 | 200 | 200 | 200 | 200 |
| **NO_3_ (µM)** | 200 | 200 | 20↑ | 200 | 20↑ | 200 |
| **NH_4_ (µM)** | 200 | 200 | 200 | 200 | 200 | 200 |
| **DIN (µM)** | 200 | 200 | 20↑ | 200 | 200 | 200 |
| **TSS (mg/l)** | 200 | 200 | 200 | 200 | 200 | 200 |
| **Chl-a (µg/l)** | 200 | 200 | 20↑ | 200 | 200 | 200 |
| **Alkalinity (meq/l)** | 200 | 200 | 200 | 200 | 200 | 200 |
| **OM decomposition** | - | 200 | 200 | - | 200 | 200 |

|  | D30 | | D90 | |  |
| --- | --- | --- | --- | --- | --- |
| Conc. (µg/L) | Vegetation cover (%) | Biomass (kg DW/m^2^) | Vegetation cover (%) | Biomass (kg DW/m^2^) | RGR (d-1) |
| 0 | 40 | 0.41 | 56 | 0.52 | 0.004 |
| 0 | 20 | 0.09 | 64 | 0.63 | 0.033 |
| 0 | 25 | 0.17 | 65 | 0.76 | 0.025 |
| 2 | 15 | 0.01 | 31 | 0.21 | 0.051 |
| 2 | 25 | 0.17 | 70 | 0.21 | 0.004 |
| 2 | 30 | 0.25 | 56 | 0.44 | 0.010 |
| 20 | 20 | 0.09 | 51 | 0.70 | 0.034 |
| 20 | 40 | 0.41 | 49 | 0.46 | 0.002 |
| 20 | 20 | 0.09 | 48 | 1.25 | 0.044 |
| 200 | 15 | 0.01 | 43 | 0.41 | 0.062 |
| 200 | 60 | 0.73 | 52 | 1.07 | 0.006 |
| 200 | 65 | 0.80 | 64 | 0.98 | 0.003 |

**Table S7**. Average values of vegetation cover and biomass of *M. spicatum* on D30 and D90. Relative growth rate (RGR) average from D30 to D90. Treatment corresponds to the different difenoconazole concentrations applied.

|  | Vegetation cover  (%) | Biomass  (kg DW m-2) | RGR (d-1) |
| --- | --- | --- | --- |
| D30 | 0.667 | 0.668 | 0.988 |
| D90 | 0.112 | 0.105 |  |

**Table S8**. Results of ANOVA (p-values) for cover and biomass of *M. spicatum* on D30 and D90. Relative growth rate (RGR) average from D30 to D90.


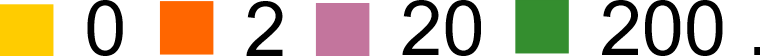


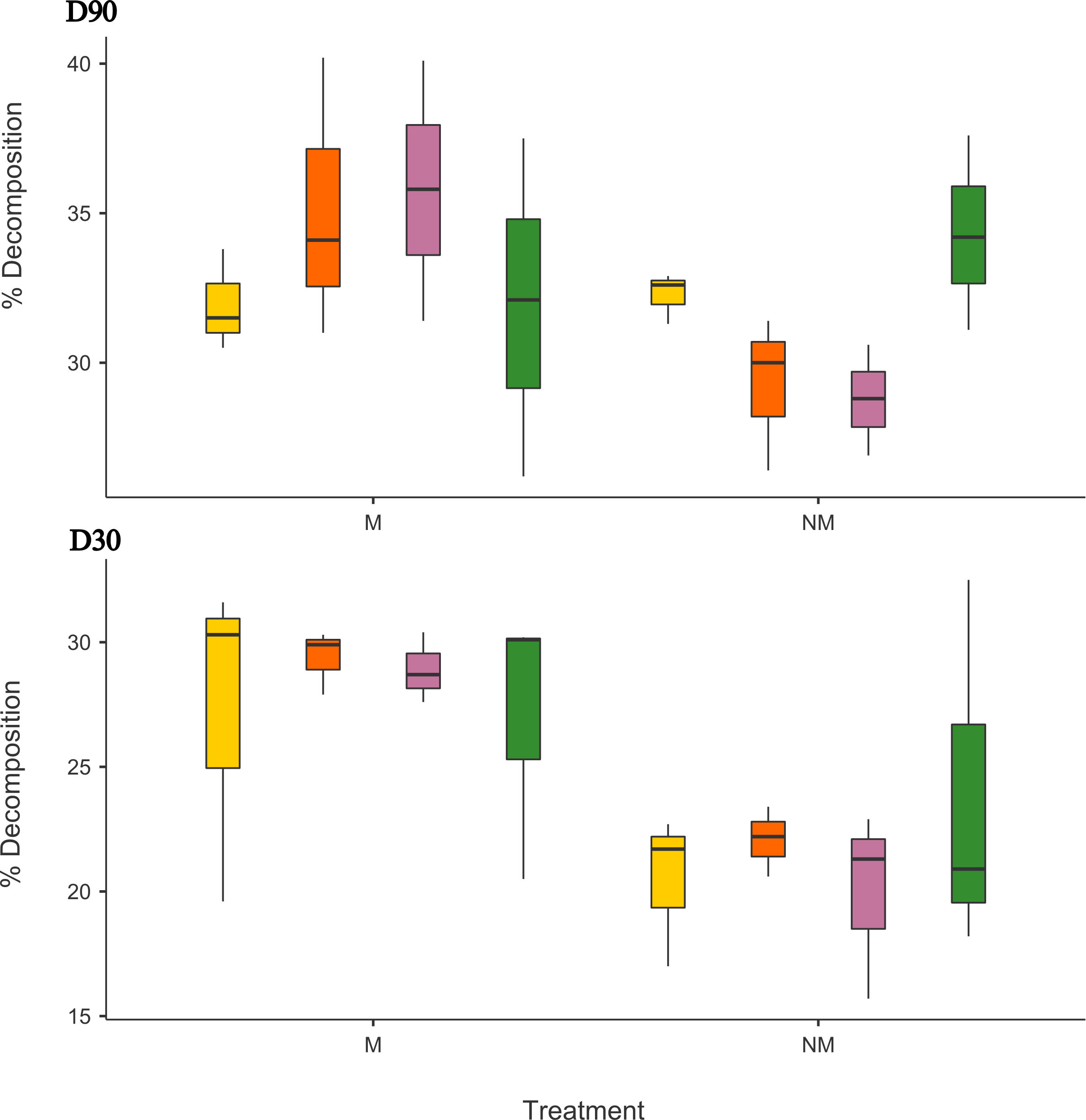


**Figure S1**. Box plots showing the distribution of organic matter decomposition in the different difenoconazole concentrations and ecological condition (M: macrophytes; NM: no macrophytes) on D30 and D90.

**Table S9.** Average values of abundance (N), species richness (S) and Shannon diversity index (H’) for the zooplankton and macroinvertebrate communities in each treatment. M: Macrophytes treatment; NM: Non-macrophytes treatment. Abundance for zooplankton is expressed in individuals/L, and for macroinvertebrates in individuals/sample. On D60, the macroinvertebrate community was not sampled.

|  |  |  | Zooplankton | | | Macroinvertebrates | | |
| --- | --- | --- | --- | --- | --- | --- | --- | --- |
|  |  |  | N | S | H´ | N | S | H´ |
| D-7 | M | 0 | 112.33 | 2.47 | 1.21 | 102.67 | 2.16 | 1.46 |
|  |  | 2 | 414.67 | 1.98 | 1.30 | 120.00 | 2.52 | 1.80 |
|  |  | 20 | 305.00 | 2.25 | 1.22 | 162.33 | 2.12 | 1.43 |
|  |  | 200 | 180.33 | 1.93 | 1.53 | 192.67 | 2.31 | 1.39 |
|  | NM | 0 | 215.67 | 2.82 | 1.85 | 73.33 | 2.02 | 1.57 |
|  |  | 2 | 255.33 | 2.17 | 1.82 | 102.33 | 1.65 | 1.41 |
|  |  | 20 | 292.00 | 2.68 | 1.62 | 51.67 | 2.29 | 1.64 |
|  |  | 200 | 198.33 | 1.95 | 1.45 | 111.67 | 1.84 | 1.26 |
| D30 | M | 0 | 117.67 | 2.44 | 1.67 | 94.33 | 2.20 | 1.76 |
|  |  | 2 | 268.33 | 2.05 | 1.22 | 133.67 | 2.06 | 1.44 |
|  |  | 20 | 117.33 | 2.20 | 1.24 | 113.00 | 1.70 | 1.60 |
|  |  | 200 | 10.00 | 2.92 | 1.44 | 126.67 | 1.73 | 1.26 |
|  | NM | 0 | 349.67 | 1.90 | 1.46 | 102.67 | 1.91 | 1.44 |
|  |  | 2 | 574.33 | 1.62 | 1.48 | 136.33 | 1.76 | 1.47 |
|  |  | 20 | 543.00 | 1.95 | 1.48 | 125.00 | 1.51 | 1.18 |
|  |  | 200 | 10.33 | 2.87 | 1.63 | 114.33 | 1.48 | 1.17 |
| D60 | M | 0 | 64.67 | 3.04 | 1.62 | - | - | - |
|  |  | 2 | 160.00 | 2.44 | 1.11 | - | - | - |
|  |  | 20 | 54.00 | 2.72 | 1.70 | - | - | - |
|  |  | 200 | 56.00 | 1.99 | 1.31 | - | - | - |
|  | NM | 0 | 398.67 | 2.11 | 1.32 | - | - | - |
|  |  | 2 | 193.33 | 2.02 | 1.35 | - | - | - |
|  |  | 20 | 314.67 | 1.49 | 1.07 | - | - | - |
|  |  | 200 | 15.33 | 2.81 | 1.63 | - | - | - |
| D90 | M | 0 | 149.33 | 1.95 | 1.07 | 137.67 | 2.17 | 1.86 |
|  |  | 2 | 216.33 | 2.44 | 1.15 | 151.00 | 2.07 | 1.94 |
|  |  | 20 | 170.00 | 2.11 | 1.12 | 137.00 | 2.19 | 1.91 |
|  |  | 200 | 56.00 | 1.72 | 0.87 | 87.33 | 1.91 | 1.76 |
|  | NM | 0 | 435.33 | 1.16 | 1.24 | 96.00 | 2.07 | 1.94 |
|  |  | 2 | 275.33 | 2.30 | 1.53 | 120.67 | 2.29 | 1.78 |
|  |  | 20 | 370.67 | 1.31 | 1.20 | 67.00 | 1.92 | 1.73 |
|  |  | 200 | 123.33 | 2.14 | 1.39 | 91.00 | 1.74 | 1.70 |

**Table S10.** Results of ANOVA (p-values) for abundance (N), species richness (S), and Shannon diversity index (H’) for the zooplankton and macroinvertebrate communities in each treatment. Macrophytes indicate the effect of the two ecological conditions. Difenoconazole indicates the effect of the chemical concentrations, and the Interaction indicates the interaction of both. * Indicates statistically significant effects (p-value < 0.05). D: day relative to the first difenoconazole application. On D60, the macroinvertebrate community was not sampled.

|  |  | Zooplankton | | | Macroinvertebrates | | |
| --- | --- | --- | --- | --- | --- | --- | --- |
|  |  | N | S | H' | N | S | H' |
| D-7 | Macrophytes | 0.819 | 0.157 | 0.004* | 0.001* | 0.016* | 0.728 |
|  | Difenoconazole | 0.169 | 0.02* | 0.802 | 0.034* | 0.86 | 0.569 |
|  | Interaction | 0.415 | 0.829 | 0.134 | 0.153 | 0.047* | 0.497 |
| D30 | Macrophytes | 0.028* | 0.199 | 0.313 | 0.944 | 0.116 | 0.111 |
|  | Difenoconazole | <0.01** | 0.032* | 0.437 | 0.306 | 0.135 | 0.172 |
|  | Interaction | 0.615 | 0.895 | 0.458 | 0.927 | 0.960 | 0.519 |
| D60 | Macrophytes | 0.016* | 0.144 | 0.417 | - | - | - |
|  | Difenoconazole | 0.010* | 0.688 | 0.431 | - | - | - |
|  | Interaction | 0.635 | 0.1 | 0.031* | - | - | - |
| D90 | Macrophytes | 0.563 | 0.096 | 0.025* | 0.035* | 0.476 | 0.347 |
|  | Difenoconazole | 0.063 | 0.037* | 0.586 | 0.131 | 0.163 | 0.549 |
|  | Interaction | 0.366 | 0.1 | 0.556 | 0.272 | 0.451 | 0.692 |

**Table S11**. Calculated NOECs (No Observed Effect Concentrations) for abundance (N), species richness (S), and Shannon diversity index (H’) for the zooplankton and macroinvertebrate communities in each treatment. ↑: abundance increase; ↓: abundance decrease. D: day relative to the first difenoconazole application. M: Macrophytes treatment; NM: Non-macrophytes treatment. NOECs are expressed in µ/L.

|  |  | **M** | | | | **NM** | | | |
| --- | --- | --- | --- | --- | --- | --- | --- | --- | --- |
|  |  | **D-7** | **D30** | **D60** | **D90** | **D-7** | **D30** | **D60** | **D90** |
| Zooplankton | **N** | 200 | 20↓ | 200 | 20↓ | 200 | 20↓ | 20↓ | 20↓ |
|  | **S** | 20↓ | 200 | 200 | 200 | 20↓ | 20↑ | 200 | 200 |
|  | **H'** | 200 | 200 | 200 | 200 | 200 | 200 | 200 | 200 |
| Macroinvertebrates | **N** | 200 | 200 | n.s. | 200 | 20↑ | 200 | n.s. | 200 |
|  | **S** | 200 | 200 | n.s. | 200 | 200 | 200 | n.s. | 200 |
|  | **H'** | 200 | 20↓ | n.s. | 200 | 200 | 200 | n.s. | 200 |


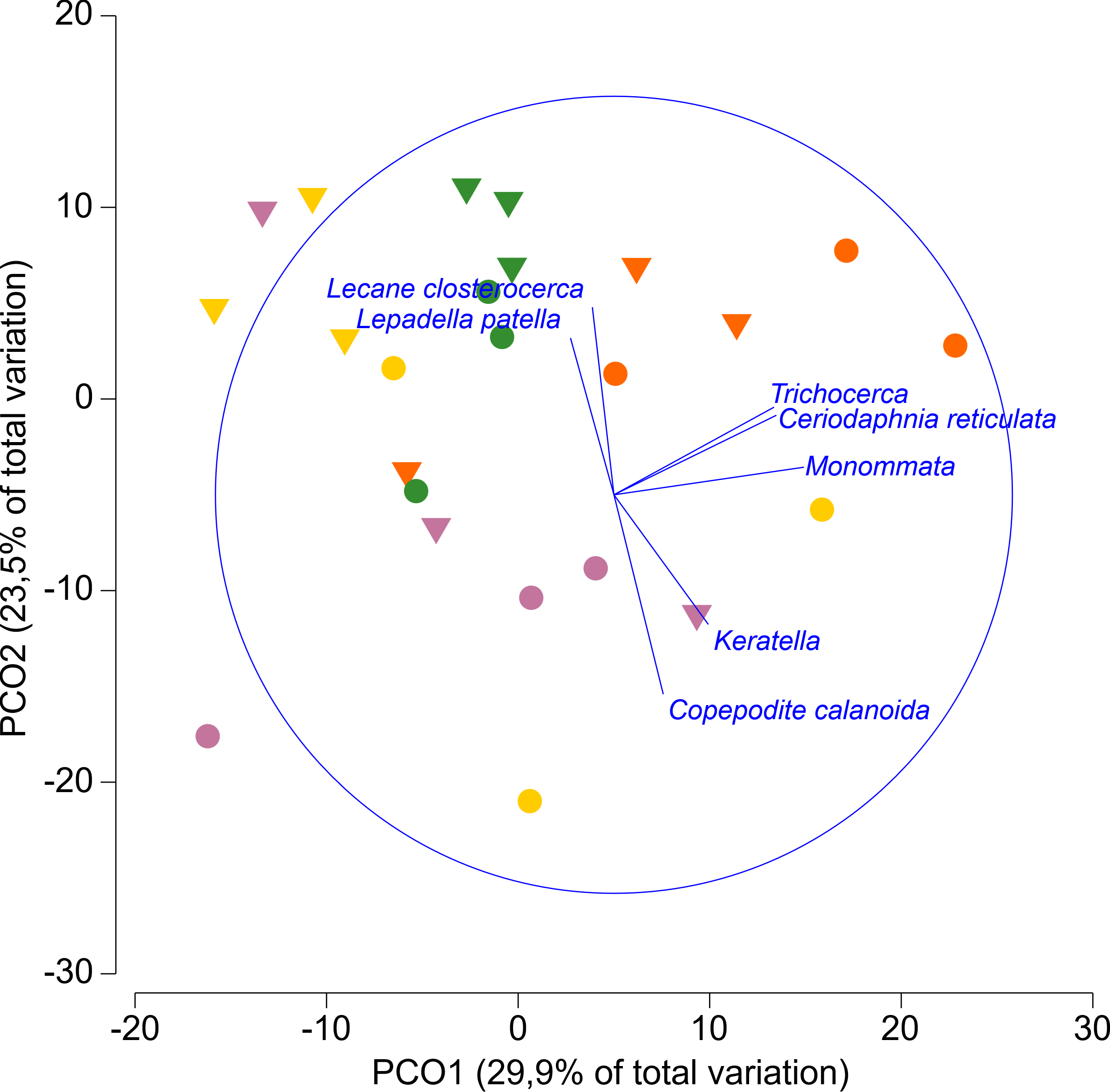


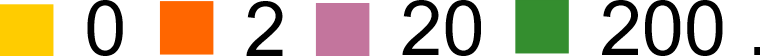


**Figure S2*.*** Principal Correspondence Analysis (PCoA) for zooplankton on D-7. The points represent all the mesocosms. The fungicide concentration is classified by colours and the two ecological conditions are represented by different symbols (●NM; ▼M). Distances between points are proportional to similarities in community composition. The species are represented in correlation with the abundance in the different mesocosms.


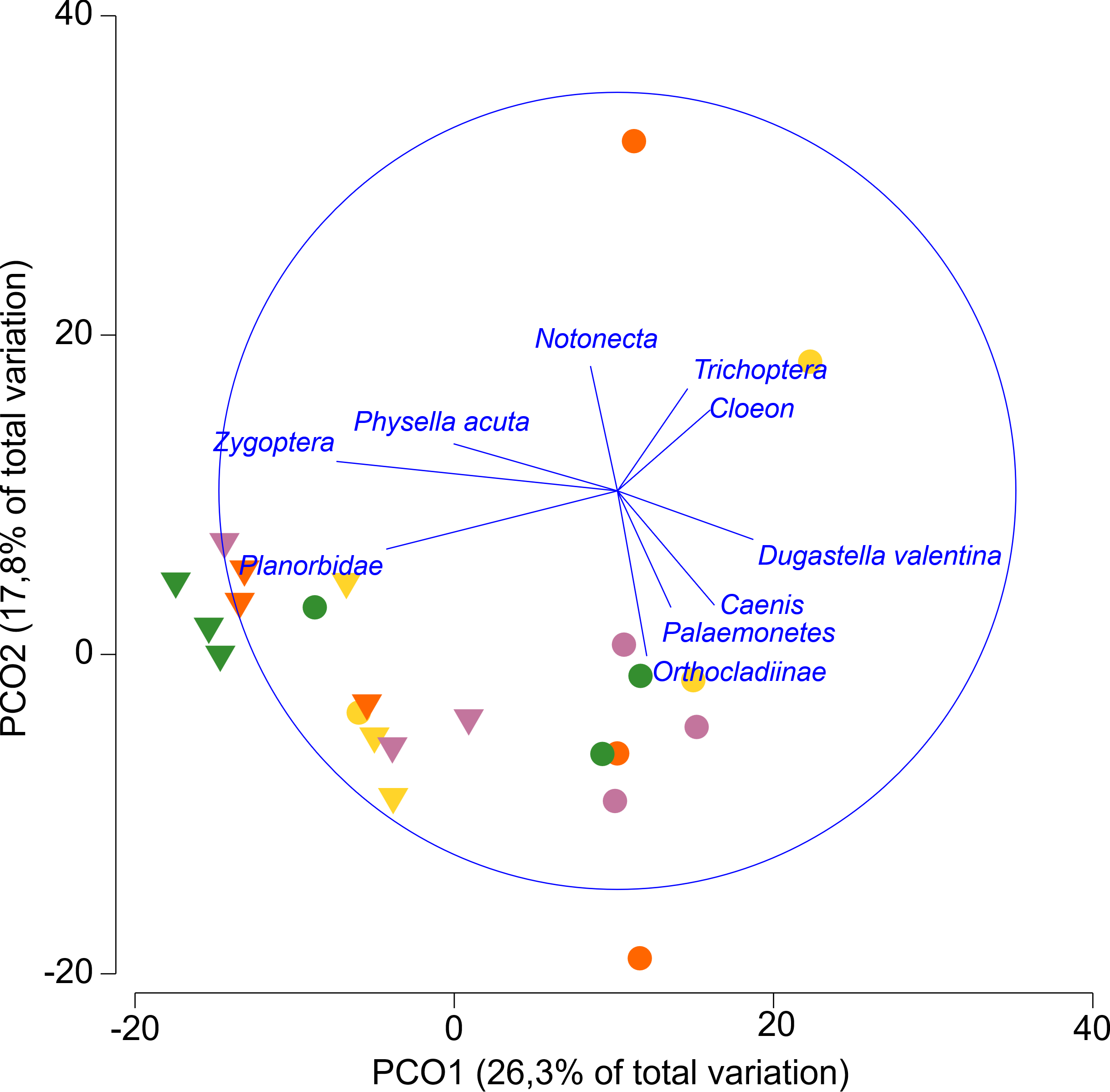


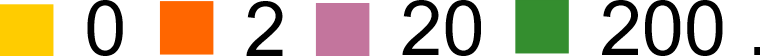


**Figure S3*.*** Representation of the first two axes of the Principal Correspondence Analysis (PCoA) for macroinvertebrates on D-7. The points represent all the mesocosms. The fungicide concentrations are classified by colours and the two ecological conditions are represented by different symbols (●NM; ▼M). Distances between points are proportional to similarities in community composition. The species are represented in correlation with the abundance in the different mesocosms.

**Table S12.** Calculated NOECs (No Observed Effect Concentrations) for the zooplankton and macroinvertebrate communities. ↑: abundance increase; ↓: abundance decrease. D: day relative to the first difenoconazole application. M: Macrophytes treatment; NM: Non-macrophytes treatment. Empty cells refer to sampling dates in which the taxon was not present, so a NOEC could not be calculated. n.s.: not sampled. NOECs are expressed in µ/L.

|  | **M** | | | | **NM** | | | |
| --- | --- | --- | --- | --- | --- | --- | --- | --- |
|  | **D-7** | **D30** | **D60** | **D90** | **D-7** | **D30** | **D60** | **D90** |
| **Zooplankton** |  |  |  |  |  |  |  |  |
| *Daphnia pulicaria* |  |  |  |  | 200 |  |  |  |
| *Ceriodaphnia reticulata* | 200 | 200 | 200 | 200 | 200 | 200 | 200 | 200 |
| *Chydorus sphaericus* |  |  | 20↓ |  |  |  | <2↓ |  |
| *Simocephalus vetulus* | 200 | 200 | 200 | 200 | 200 |  | 2↓ | 200 |
| *Pleuroxus aduncus* |  |  |  |  | 200 |  |  |  |
| *Macrothrix sp* | 200 |  |  |  | 200 |  |  |  |
| *Kurzia sp* |  |  |  |  |  |  | 200 |  |
| *Copepodite Cyclopoida* | 200 | 200 | 2↓ | 200 | 20↓ | 200 | <2↓ | 200 |
| *Copepodite Calanoida* | 200 | 200 | 200 | 20↓ | 2↑ | 200 | 200 | 200 |
| *Nauplii* | 200 | 200 | 20↓ | 20↓ | 200 | 20↓ | 20↓ | 20↓ |
| *Anuraeopsis sp* | 200 |  |  |  | 200 | 200 |  |  |
| *Bdelloidea* |  | 200 | 200 |  | 200 | 200 | 200 |  |
| *Brachionus calyciflorus* | <2↓ |  |  |  | 200 | 200 |  |  |
| *Brachionus quadridentatus* | |  |  |  |  | 200 |  |  |
| *Brachionus havanaensis* | 200 | 200 |  |  | <2↓ | 200 | 200 |  |
| *Platyas sp* |  |  |  | 200 |  | 200 |  | 20↑ |
| *Colurella sp* | 200 |  |  |  |  |  |  |  |
| *Euchlanis sp* |  |  | 200 |  |  |  | 200 |  |
| *Keratella spp.* | 200 | 20↓ | 200 | 20↓ | 200 | 20↓ | 20↓ | 200 |
| *Lecane cornuta* | 200 |  | 200 | 200 |  |  | 200 | 200 |
| *Lecane quadridentata* | 20↓ | 200 | 200 | 200 | 200 | 200 | 200 |  |
| *Lepadella patella* |  |  | 20↑ | 20↑ | 200 |  | 200 | 200 |
| *Lecane bulla* | 200 | 200 | 20↑ | 200 | 200 | 200 | 200 | 200 |
| *Lecane closterocerca* |  | 200 | 20↑ | 20↑ | 200 |  | 200 | 200 |
| *Lecane candida* |  |  |  |  |  |  |  |  |
| *Lecane luna* |  |  |  |  |  | 200 | 200 | 200 |
| *Lecane lunaris* | 200 |  | 200 |  | 200 |  |  |  |
| *Testudinella sp* | 200 | 200 | 2↓ | 200 | 200 | 200 | 200 |  |
| *Trichocerca sp* |  | 200 | 200 | 200 |  | 200 | 200 | 200 |
| *Trichotria sp* |  |  | 200 | 200 |  |  |  |  |
| *Hexarthra sp* |  |  |  |  |  | 200 | 200 | 20↓ |
| *Polyarthra dolichoptera* | 200 | 200 | 200 | 200 | 200 | 200 | <2↓ | 200 |
| *Notholca sp* |  |  |  |  |  |  | 200 |  |
| *Monommata sp* |  |  |  |  | 200 |  |  | 200 |
| *Squatinella rostrum* | 200 | 200 | 200 | 200 | 200 |  | 200 | 200 |
| *Synchaeta sp* |  |  |  |  |  |  | 200 |  |
| **Macroinvertebrates** |  |  |  |  |  |  |  |  |
| *Dugastella* | 200 | 20↓ | n.s. | 20↓ | 200 | 200 | n.s. | 20↓ |
| *Palaemonetes zariquiey* | 200 | 200 | n.s. | 20↓ | 200 | 200 | n.s. | 200 |
| *Echinogammarus* | 200 | 200 | n.s. | 200 |  |  | n.s. | 200 |
| *Isopoda* | 200 |  | n.s. |  |  | 200 | n.s. |  |
| *Caenis sp.* | 200 | 200 | n.s. | 200 | 200 | 200 | n.s. | 200 |
| *Cloeon sp.* | 200 | 200 | n.s. | 200 | 200 | 200 | n.s. | <2↓ |
| *Hydracarina* |  |  | n.s. |  |  | 200 | n.s. | 200 |
| *Zygoptera* | 200 | 200 | n.s. | 200 | 200 | 200 | n.s. | 200 |
| *Anisoptera* | 200 | 200 | n.s. | 200 | 200 | 200 | n.s. | 200 |
| *Chironomidae* | 200 | <2↑ | n.s. | 200 | 20↑ | 200 | n.s. | 200 |
| *Orthochladiinae sp.* | 200 | 200 | n.s. | 200 | 200 |  | n.s. | 20↑ |
| *Culicidae* | 200 |  | n.s. |  | 200 |  | n.s. |  |
| *Trichoptera* |  | 200 | n.s. | 200 |  | 200 | n.s. | 200 |
| *Diptera pupa* |  |  | n.s. |  |  | 200 | n.s. |  |
| *Corixa sp.* | 200 | 200 | n.s. |  | 200 | 200 | n.s. |  |
| *Gerris sp.* |  | 200 | n.s. | 200 | <2↓ |  | n.s. |  |
| *Notonecta sp.* | 200 | 20↑ | n.s. | 200 | 200 | 20↑ | n.s. | 200 |
| *Mesoveliidae* | 20↑ |  | n.s. |  | 200 |  | n.s. |  |
| *Dytiscidae adult* | 200 | 200 | n.s. | 200 | 200 | 200 | n.s. | 200 |
| *Dytiscidae larvae* | 200 |  | n.s. |  |  |  | n.s. |  |
| *Hydroglyphus* | 200 | 20↓ | n.s. | 200 | 200 | 200 | n.s. | 200 |
| *Physella acuta* | 200 | 200 | n.s. | 200 | 200 | 200 | n.s. | 200 |
| *Melanopsis* | 200 | 200 | n.s. | 200 | 20↑ | 20↑ | n.s. | 200 |
| *Planorbidae* | 200 | 20↓ | n.s. | 2↓ | 200 | 200 | n.s. | 200 |
| *Platyhelminthes* | 200 | 200 | n.s. | 200 | 200 |  | n.s. |  |
| *Araneae* |  |  | n.s. | 200 |  |  | n.s. | 200 |
